# Preference of homebirth and associated factors among pregnant women in Arba Minch health and demographic surveillance site, Southern Ethiopia

**DOI:** 10.1371/journal.pone.0276682

**Published:** 2022-10-27

**Authors:** Solomon Seyife Alemu, Teklemariam Gultie Ketema, Kassahun Fikadu Tessema, Jira Wakoya Feyisa, Awol Arega Yimer, Birhanu Negese Kebede

**Affiliations:** 1 Departments of Midwifery, College of Health Sciences, Mettu University College of Health Science, Mettu, Ethiopia; 2 Departments of Midwifery, College of Medicine and Health Sciences, Arba Minch University, Arba Minch, Ethiopia; 3 Department of Public Health, Institute of Health Sciences, Wollega University, Nekemte, Ethiopia; University of Mississippi Medical Center, UNITED STATES

## Abstract

**Background:**

Home birth preference is the need of pregnant women to give birth at their home with the help of traditional (unskilled) birth attendants. Homebirth with unskilled birth attendants during childbirth is the main leading indicator for maternal and newborn death. In Ethiopia, numbers of women prefer homebirth which is assisted by unskilled personal. However, there is no information regarding the problem in the Arba Minch zuria woreda. Therefore, it is important to identify prevalence of preference of homebirth and associated factors.

**Objectives:**

This study aimed to assess the preference of home birth and associated factors among pregnant women in Arba Minch health and demographic surveillance site.

**Method and materials:**

A community-based cross-sectional study was conducted among pregnant women in Arba Minch health and demographic surveillance site, from May 1 to June 1, 2021. Using simple random sampling technique, 416 study samples were selected. Data were collected by interviewer-administered questionnaire. Data were coded and entered into Epi-Data version 4.4.2.1 computer software and exported to Statistical Package for Social Sciences software version 25 for analysis. Bi-variable binary logistic regression for the selection of potential candidate variables at p-value < 0.25 for multivariable analysis and multivariable binary logistic regression to identify the association between homebirth preference and independent variables were carried out. The level of statistical significance was declared at a p-value < 0.05.

**Result:**

In this study, in Arba Minch demographic health surveillance site, the prevalence of preference of pregnant women to give birth at their home was 24% [95%CI: (19.9%-28.2%)] The factors significantly associated with the preference of home birth were husband involvement in decision making [AOR: 0.14 (0.05–0.38)], no access of road for transportation [AOR: 2.4 (1.2–5.18)], not heard about the benefit of institutional birth [AOR: 5.3 (2.3–12.2)], poor knowledge about danger signs [AOR: 3 (1.16–7.6)], negative attitude toward services [AOR: 3.1 (1.19–8.02)], and high fear to give birth at institution [AOR: 5.12 (2.4–10.91)].

**Conclusions:**

In Arba Minch demographic health surveillance site, the prevalence of preference of pregnant women to give birth at their home was 24%. Husband involvement in decision making, no access of road for transportation, not heard about the benefit of institutional birth, poor knowledge about danger signs, negative attitude toward services, and high fear to give birth at health institutions were factors significantly associated with the preference of home birth.

## Introduction

According to the report in 2017, the coverage and progress of skilled birth attendants from the year 2012–2017 varied across the world, for instance, 54% in sub-Saharan countries versus 98% in Eastern Europe countries [1, 2]. In Ethiopia, the recent report shows that 52% of childbirths occurred at home with a lack of skilled birth attendants [3]. More than 50% of the risk of maternal mortality and 75% of stillbirth are reduced by providing emergency obstetrics care during labor and childbirth [4, 5]. However, in 2017 unevenly 295,000 women died due to pregnancy complications, labor and delivery and post natal complications more than 94% of this death occurred in low-income countries [6]. Ethiopia is one of the sub-Saharan countries with a high prevalence of maternal mortality ratio that accounts for 412 per 100,000 live births [7].

Different literature showed that women prefer home birth hence thinking childbirth at home is more comfortable, safer, and gives greater self-control than the health facilities [8, 9]. However, home birth in developing countries is attended by unskilled personal or family members with a lack of infrastructure [5]. Thus, home birth is difficult for early detection and management of complications like a failure of progress of labor, obstructed labor, postpartum hemorrhages, convulsion, infection, fetal distress, and others [8]. Even with highly trained health professionals, home birth is not safe in some conditions like heart disease, renal disease, diabetes, preeclampsia, hemorrhage, prior cesarean section delivery, and active genital warts [10].

In most circumstances, women attend health institutions after trial of labor and birth at their home [9]. The common reasons for attending health facilities are complications like retained placenta, excessive vaginal bleeding, shock, third and fourth-degree tear, cervical tear, uterine rupture, and severe anemia with the need for blood transfusion. These complications increase morbidity and mortality of the women [8, 9]. In addition to this, it increases the burden on health facilities and health professionals to manage the complications with limited resources [10].

In Ethiopia, the effort taken to reduce maternal and neonatal death inclusive of; providing free maternal service including, labor and delivery, extending health extension workers, health post and referral system [10]. Age of the pregnant mothers, lack of knowledge on danger sign, poor road access, lack of ANC follow-up, low household income, place of last delivery, parity, and low educational status contributes to the preference of home birth [11–13].

The global community experts plan to overcome the challenges faced in the millennium development goals to sustainable development. By 2030 they aimed to decrease maternal mortality to 70 per 100,000 and neonatal mortality to 12 per 1000. These new strategies considered the shortage of resources and skilled personnel [14].

Safe delivery service is one of the crucial maternity care issues for pregnant women. It is necessary to find out the factors that affect care-seeking behavior in a given context. The majority of pregnant women in developing countries do not decide on a place of birth before the onset of labor. Women may give birth at health institutions without their preference by shifting their plans due to complications that occur during labor and delivery. In our country, most of the previous studies were conducted on institutional birth utilizations, not on their preference. The studies conducted in Ethiopia at Jimma town southwest, South Tigrai zone, Debre Tabor, and Debre Markos showed that the numbers of urban pregnant women prefer home birth. However, as far as the investigators knowledge is concerned there was no study done on the preference of home birth among pregnant women in rural areas. Additionally, this study addressed different variables (transportation facilities, benefits of institutional delivery, fear of child birth at institution) those were not addressed by previous studies.

## Methods and materials

### Study design and study area

A community-based cross-sectional study was conducted in Arba Minch Health and Demographic Surveillance Site. Arba Minch Health and Demographic Surveillance Site are located in Arba Minch Zuria and Gacho Baba districts, Gamo Zone, Southern Ethiopia, 500 km to the South of Addis Ababa, the capital city of Ethiopia. Arba Minch Zuria district and Gacho Baba district had a total of 31 kebeles [smallest administrative units] and it is included under Arba Minch Zuria Demographic and Health Development Program (AM-DHDP). AM-DHDP is owned by Arba Minch University and it is one of the six public universities Health and Demographic Surveillance System (HDSS) in Ethiopia. The surveillance site consists of nine kebeles which were selected in the representation of 31 kebeles in the district. From them, 6 kebeles were found in Arba Minch zuria district, and the rest three were found in Gacho baba districts. Farming is the predominant occupation of residents in the districts. Based on the 2007 census projection, the districts had a total population of 164,529. The district has 7 health centers and 37 health posts [15]. Around 81.8% of women gave birth at home in Arba Minch Zuria district [16].

### Data collection period

Data were collected from May 1- June 1, 2021 from randomly selected pregnant women of Arba Minch zuria woreda.

### Study population

Pregnant women living in selected nine Kebeles of Arba Minch health and demographic surveillance site were study population for this study.

### Inclusion criteria

Pregnant women living in Arba Minch health and demographic surveillance site included in the study.

### Exclusion criteria

Pregnant women with severely illness as well as those who were in labor during data collection period were excluded.

### Sample size determination and sampling technique

The sample size was determined by using a single population proportion formula, by considering the following assumptions; taking a proportion of home birth preference conducted in Simada district Ahmara region, Ethiopia, 56.4% proportion, 95% confidence level and power 80 considering 10% non-response rates [11].

$$\text{n}=[{\left(\text{Z}\alpha /2\right)}_{2}\;\text{p}\left(1-\text{p}\right)]/{\text{d}}_{2}=\underline{378}\;\text{by}\;\text{adding}\;10\;\%\;\text{non-response}\;\text{rate}\;\text{the}\;\text{final}\;\text{sample}\;\text{size}=\underline{416}$$

**Where**;

n = the desired sample size.

Zα/2 = Standard normal deviate of 1.96 which corresponds to 95% confidence level (z value at Alpha = 0.05).

P = Proportion of home birth (56.4%).

d = an absolute precision (margin of error0 which is 5%.

Hence study was conducted in Arba Minch health and demographic surveillance site registration and identification of the women becoming pregnant with their address is one of the core and continuum activities of the health extension workers assigned to the woreda. Since study was conducted in Arba Minch health and demographic surveillance site, the list of pregnant women was obtained from health extension workers working in Arba Minch health and demographic surveillance site. The total number of pregnant women obtained from health extension workers from nine kebeles of Arba Minch health and demographic surveillance site were 610. Before the selection of study participants, proportions to size allocations to each kebele were done. From the list, the required sample size (416) was selected by simple random sampling using computer-generated numbers from each kebele as per the proportions to size allocation to each kebele.

### Data collection procedure

The data were collected by using structured interviewer-administered questionnaire. The questionnaires contain questions about socio-demographic characteristics, service-related, obstetrical characteristics, knowledge on danger signs, attitude toward skilled birth services, and fear of childbirth at a health institution. These questionnaires were adapted and developed from published related literatures [11, 12, 17–20]. Nine Health and demographic surveillance site data collectors and three supervisors were used. The data were collected using interviewer-administered questionnaire with participants at their homes. Preference of homebirth was obtained from the question asked to pregnant women; “where do you prefer to give birth?” Response to this question was either of home birth or health facility [hospital, health Centre/clinic, health post, and private hospital/clinic] [11].

### Data quality control

To assure the data quality the questionnaires were translated from English to Amharic and retranslated to English for a consistent and proper check. The pre-test was done on a sample of 21 pregnant women (5% of sample size) in Mirab Abaya woreda southern part of Ethiopia. The internal consistency of the tool was assessed by a reliability test (Cronbach’s alpha). The values of Cronbach’s alpha were 0.743, 0.841, and 0.919 for knowledge, attitude, and fear of childbirth at institution questions respectively. Two days of training on data collection procedures, and the objectives of the study for data collectors and supervisors were provided. Collected data was checked for completeness on daily basis by data collectors and supervisors.

#### Study variables

*Dependent variable*. Preference of home birth. “Preference of home birth” was the dependent variable and was obtained from the question, “Where do you prefer/need to give birth [choices]?” Response to this question was prefer/need to give birth at home or at government hospital/health center or private hospital/clinic. It was then dichotomized to into prefer health facility birth = 0 and prefer home birth = 1 where respondent’s preference/need to give birth at home “prefer home birth” and all the other categories were grouped as “prefer health facility birth” [11].

*Independent variables*. The independent variables considered in this study were age of the women, marital status, ethnicity, religion, women educational status, women occupation, husband educational status, husband occupation, household income, residence, family size, Gravid, pregnancy desire, last place of delivery, last mode of delivery, last birth complication, current ANC status, number of ANC follow up, birth interval, distance from health services/facility, road access for transportation to health institutions, information on the benefit of institutional birth, Knowledge of danger signs, attitude toward skilled birth services, decision-making, and fear of childbirth at the institution.

*Operational definitions*. **Women’s fear of childbirth at health institution**: A total of 13 items were presented to assess fear of childbirth at the health institution. Women responded to their level of fear for each item by a 4-point Likert scale. The women were classified as high fear if they scored mean value and above, and low fear if they scored less than mean value to question assessing fear of childbirth at institutions [19].

**Knowledge about danger signs of pregnancy, labor, and following childbirth**: Knowledge about danger sign was assessed based on the women’s response to eight knowledge questions. Thus, women’s were considered as they have good knowledge if they answered correctly to four or more knowledge question [20].

**Women’s Attitude about skilled birth services**: A total of 7 questions were used to assess attitude. Women responded to each question in the form of very agree, agree, disagree, and very disagree. Very agree and Agree was labeled as value "1", and disagree and very disagree was as assigned value "0". Women were considered as they have positive attitudes if all questions were labeled a value "1", and negative attitudes if any of the questions are labeled "0" [17].

### Data processing and analysis

The collected data were coded and entered into Epi-Data version 4.4.2.1 software and exported to SPSS statistical software version 25 for data cleaning and further analysis. Errors related to the inconsistency of data were checked and corrected during data cleaning. Descriptive statistical analyses such as simple frequencies, percentage, median and interquartile range were used to describe the characteristics of participants.

The binary logistic regression model was fitted to identify factors associated with preference of homebirth after checking assumptions. Multi co-linearity by co-linearity matrix among the independent variables was checked. Bi-variable logistic regression analysis was performed between preference of homebirth and each of the independent variables, in sequence. Variables having a p-value of <0.25 in bi-variable logistic regression were a potential candidate for multivariable logistic regression analysis to control confounders in regression models. Variables having a p-value of less than 0.05 in the multivariable logistic regression model were considered as statistically significant. The final model was fitted with Hosmer and Lemeshow (p-value = 0.966). The strength of association between the preference of homebirth and independent variables were reported by using the adjusted odds ratio (AOR) with 95% CI.

### Ethical consideration

An ethical clearance letter was obtained from Arba Minch University, college of medicine, and health sciences research review board in 25/03/2021 with reference number IRB/1071/21. Written Permission was sought from the Health and demographic surveillance site, Arba Minch zuria and Gacho Baba districts. Written consent was obtained from each study participant before data collection and the purpose of the study was explained to the respondents. To protect confidentiality names and personal identification were not included in questionnaires. During data collection at the end of each interview women who prefer home birth were advised about the risk of home delivery. The issue of worldwide COVID 19 preventive approaches like social distancing face masks and hand sanitizer was practiced during data collection.

## Results

### Socio-demographic characteristics of the study participants

In this study, four hundred eight pregnant women volunteered to give information making a response rate of 98%. The median ages of the respondents were 29 years [interquartile range [(IQR) = (24–34)]. Regarding educational status, 165(40.4%) of the study participants and 173(45.3%) of respondents’ husbands were unable to read and write. Concerning occupational status, 244(59.8%) of study participants were housewives, and 156(40.8%) of their partners were a farmer. Eighty-eight point five percent of the participants were living in rural Kebeles. In terms of monthly household income, approximately half 188(46.1%) of the respondents earn <1000birr per month (Table 1).

**Table 1:** Socio-demographic characteristics of the study participants in Arba Minch health demographic surveillance site in May 2021.

Variables	Category	Frequency	Percent [%]
Age women in years categories	18–19	46	11.3
	20–24	69	16.9
	25–29	107	26.2
	> = 30	186	45.6
Marital status	Married	382	93.6
	Single	19	4.7
	Separated	7	1.7
Ethnicity	Gamo	312	76.5
	Gofa	31	7.6
	Walayita	52	12.7
	Gurage	11	2.7
	Others *	2	0.5
Religion	Protestant	198	48.5
	Muslim	19	4.7
	Orthodox	183	44.8
	Others **	8	2
Women educational status	Unable to read and write	165	40.4
	Primary education	121	29.7
	Secondary education	80	19.6
	Diploma and above	42	10.3
Women occupation	Housewife	244	59.8
	Government employee	33	8.1
	Merchant	40	9.8
	Private employee	39	9.6
	Student	29	7.1
	Daily labor	12	2.9
	Others***	11	2.7
Husband educational status	Unable to read and write	173	45.3
	Primary education	79	20.7
	Secondary education	77	20.2
	Diploma and above	53	13.8
Husband occupation	Government employee	44	11.6
	Merchant	62	16.2
	Farmer	156	40.8
	Daily labor	52	13.6
	Private employee	54	14.1
	Student	11	2.9
	Others***	3	0.8
Residence	Rural	361	88.5
	Urban	47	11.5
Number of household members	1–5	311	76.2
	Above 5	97	23.8
Household monthly income[ETB]	<1000	188	46.1
	1001–2000	98	24
	> = 2001	122	29.9

### Social and service-related characteristics

In this study, more than half of pregnant women 225 (55.1%) decided place of birth with their husbands. Regarding the accessibility of maternal health services, 353(86.5%) of pregnant women had reported the distance of health facilities from their residences was less than 5km (Table 2).

**Table 2:** Social and service-related characteristics of study participants in Arba Minch health demographic surveillance site, May 2021.

Variables	Category	Frequency	Percent [%]
Decision on the choice of place of birth	Women herself only	104	25.5
	Both women and her husband	225	55.1
	Only her husband	59	14.7
	Traditional birth attendants	3	0.5
	Her mother	17	4.2
Accessibilities of the road for transportation to ward health institution	Yes	254	62.3
	No	154	37.7
Estimated distance from Health institution [km]	< = 5km	353	86.5
	>5km	55	13.5

### Obstetric characteristics of the study respondents

From obstetrical characteristics of respondents, almost near to three-fourth of respondents were multigravidas. One hundred eighty-nine participants gave birth to their last child at health institutions. Regarding the current pregnancy, 72.3% of respondents reported that their current pregnancy was wanted, and 69.1% of pregnant women have antenatal care follow-up for the current pregnancy. Sixty percent reported that they heard information about the benefits of institutional delivery and 28.7% of respondents reported at least one dangerous symptom during the current pregnancy (Table 3).

**Table 3:** Obstetrical characteristics of study participants in Arba Minch health demographic surveillance site, May 2021.

Variables	Category	Frequency	Percent
Gravida	1	114	27.9
	2–5	242	59.3
	>5	52	12.8
The interval between this pregnancy and the last pregnancy	< = 1 year	15	5.1
	2 -4years	218	74.2
	> = 5 years	61	20.7
Last place of birth	Institution	189	64.3
	Home	105	35.7
Mode of delivery in last birth	SVD	240	81.6
	Assisted	30	10.2
	CS	24	8.2
Maternal complications in last childbirth	Yes	57	19.4
	No	237	80.6
The desire of this pregnancy	Wanted	295	72.3
	Unwanted	113	27.7
Gestational age of this pregnancy [month]	< = 3months	25	6.1
	4–6 months	102	25
	> = 7 months	281	68.9
ANC follow up for this pregnancy	Yes	282	69.1
	No	126	30.9
Number of ANC visits	Once–Three	190	67.4
	Four and above	92	32.6
Ever advised on the benefit of institutional birth during ANC	Yes	234	83
	No	48	17
Heard about the benefit of institutional	Yes	245	60
	No	163	40
Danger signs on this pregnancy	Yes	117	28.7
	No	291	71.3

### Pregnant women personal related characteristics

#### Knowledge about danger signs of pregnancy, labor, and following childbirth

**Fig 1:**
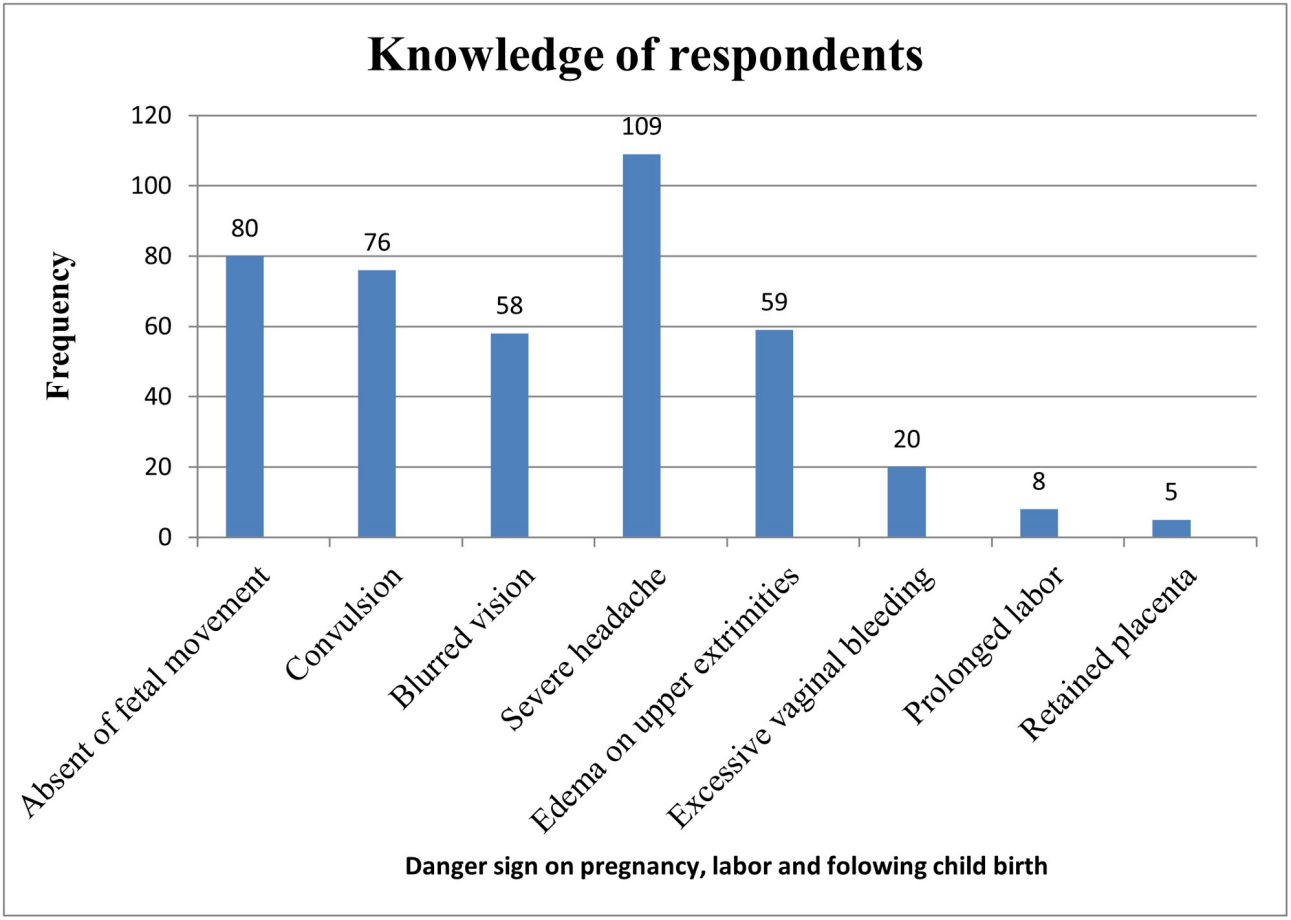
Distribution of knowledge on danger signs of pregnancy, labor, and following childbirth among pregnant women in Arba Minch health demographic surveillance site, 2021.

Concerning the knowledge about danger signs, 201(49.3%) of the participants had good knowledge about danger signs. Severe headaches, absent of fetal movement, and loss of consciousness were the danger signs mainly reported by the participants (Fig 1).

#### Attitude toward skilled care services and fear of childbirth at the institution

Among respondents, 343(84.1%) pregnant women had a positive attitude toward skilled birth services. Regarding fear of childbirth at institutions, 229(56.1%) of the study participants had less fear of childbirth at health institutions.

### Pregnant mothers place of birth preference

**Fig 2:**
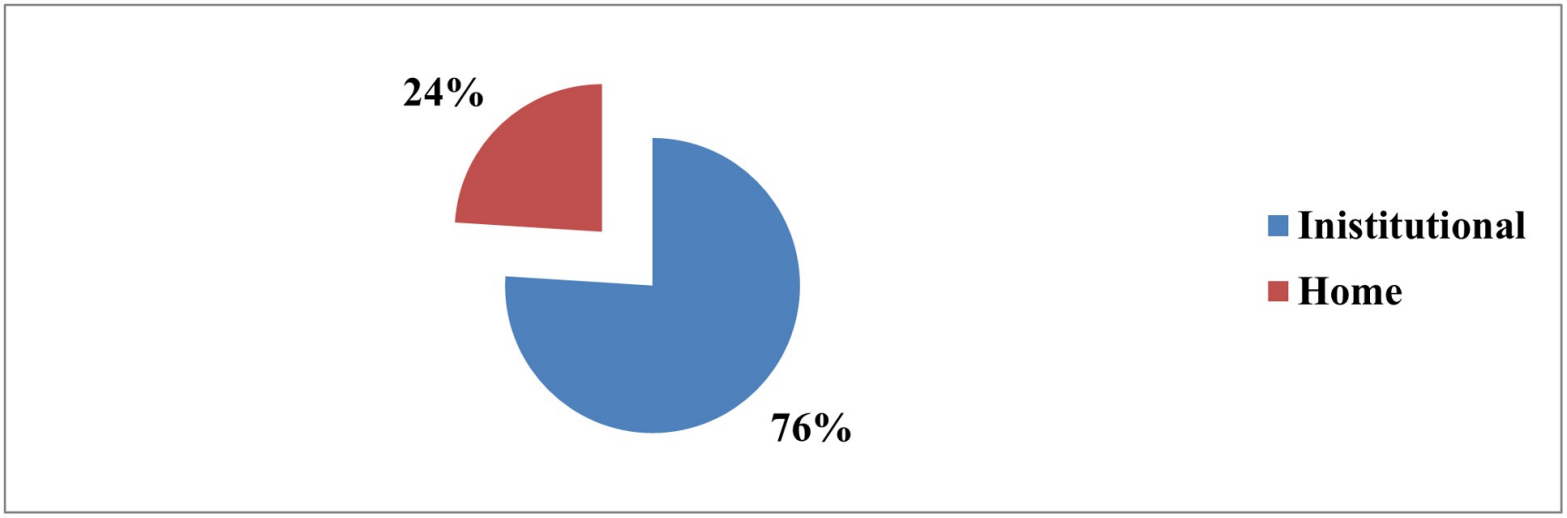
Distribution of pregnant women place birth preference in Arba Minch health demographic surveillance site May 2021.

In this study 24% [95%CI: (19.9%, 28.2%)] of pregnant women were prefer home birth (Fig 2).

### Factors associated with home birth preference

In this study, 10 variables were candidate for multivariable analysis (Table 4). After controlling for potential confounders six variables; decision making on birthplace, road access for transportation to health institution, benefit of institutional delivery, knowledge about danger signs, the attitude toward skilled birth services, and fear of childbirth at health institution significantly associated with the preference of home delivery.

**Table 4:** Bi-variable and multivariable logistic regression analysis of the home birth preference among pregnant women in Arba Minch health demographic and surveillance site May 2021.

Variables	Category	Place birth preference		Crude odds ratio [95% CI]	Adjusted odds ratio 95%CI]	P-Value
		Home[98]	HI[310]			
Age of the women	18–19	9[9.2%]	37[11.9%]	0.63[0.28–1.39]	0.48[0.14–1.7]	0.245
	20–24	11[11.2%]	58[18.7%]	0.49[0.24–0.97]	0.64[0.2–1.92]	0.423
	25–29	26[26.5%]	81[26.1%]	0.83[0.48–1.43]	1.3[0.54–3.12]	0.556
	> = 30	52[53.1%]	134[43.3%]	1		
Educational status of women	Unable read and write	62[63.3%]	103[33.2%]	5.52 [2.8–10.83]	1.58[0.58–4.37]	0.375
	Primary	24[24.5%]	97[31.3%]	2.27[1.08–4.78]	1.12[0.36–3.50]	0.845
	Secondary and above	12[12.2%]	110[35.5%]	1	1	
Marital status	In union	80[81.6%]	302[97.4%]	1	1	
	Not in union	18[18.4%]	8[2.6%]	8.49[3.56–20.24]	2.34 [0.7–7.84]	0.168
Income of household per month[Birr]	< = 1000	60[61.2%]	128[41.3%]	4.730[2.37–9.44]	1.7[0.59–4.93]	0.326
	1000–1999	27[27.6%]	71[22.9%]	3.84[1.79–8.22]	1.77[0.5–6.03]	0.358
	> = 2000	11[11.2%]	111[35.8%]	1	1	
Road access for transportation to reach health institution	Yes	35[35.7%]	219[70.6%]	1	1	
	No	63[64.3%]	91[29.4%]	4.33[2.68–7]	2.4[1.2–5.18]	0.024*
Decision making on the place of birth	Women her self	38[38.7%]	66[21.3%]	1		
	Both her and her husband	13[13.3%]	212[68.4%]	0.11[0.05–0.21]	0.14[0.05–0.38]	0.001*
	Husband, TBA, and her mother	47[48%]	32[10.3%]	2.55[1.4–4.65]	1.55[0.64–3.74]	0.329
Heard about the benefit of institutional delivery	Yes	16[16.3]	229[73.9%]	1	1	
	No	82[83.7%]	81[26.1%]	14.5[8.01–26.21]	5.3[2.3–12.2]	0002*
Knowledge of women’s on danger sign	Good knowledge	10[10.2%]	191[61.6%]	1	1	
	Poor knowledge	88[89.8%]	119[38.4%]	14.12[7.06–28.24]	3[1.16–7.6]	0.024*
The attitude of the respondents	Positive attitude	63[35.7%]	280[9.7%]	1	1	
	Negative attitude	35[64.3%]	30 [90.3%]	5.19[2.97–9.07]	3.1[1.19–8.02]	0.020*
Fear of childbirth at the institution	High fear	79[80.6%]	100[32.3%]	8.73[5.02–15.20]	5.12[2.4–10.91]	0.001*
	Less fear	19[19.4%]	210[67.7%]	1	1	

The odds of preference of home birth among pregnant women who decided birthplace with their husbands were 86% [AOR: 0.14 (0.05–0.38)] less likely compared to pregnant women who decided alone. Similarly, the odds of preference of home birth among pregnant women who have no road access for transportation were 2.4 times [AOR: 2.4 (1.2–5.18)] higher compared to those who have road access for transportation. The benefit of institutional birth was one of the factors significantly associated with the preference of home birth. The odds of preference of home birth among pregnant women who did not hear about the benefit of institutional birth were 5.3 times [AOR: 5.3 (2.3–12.2)] higher compared to those who heard about the benefit of institutional delivery. Knowledge about danger signs was significantly associated with the preference of home birth. The odds of preference of home of birth among pregnant women who had poor knowledge of danger signs were 3 times [AOR: 3 (1.16–7.6)] higher compared to pregnant women who had good knowledge on danger signs.

Attitude towards skilled birth services is also the other factor that is significantly associated with the preference of home birth. The odds of preference of home birth among pregnant women who had a negative attitude toward the skilled birth services were 3.1 times [AOR: 3.1 (1.19–8.02)], higher when compared with pregnant women who had a positive attitude toward the skilled birth services. The other significant variable was fear of childbirth at health institutions. The odds of preference of home birth among pregnant women who had high fear of childbirth at health institutions were 5.1 times [AOR: 5.12 (2.4–10.91)] higher compared to pregnant women who had less fear of childbirth at health institutions (Table 4).

## Discussion

This study assessed the preference of home birth and associated factors among pregnant women in Arba Minch Health and demographic surveillance site, southern Ethiopia, 2021. Decision-making on birthplace, road accessibilities to health institutions, benefit of institutional delivery, knowledge about danger signs, the attitude toward skilled birth services, and fear of childbirth at health institutions were significantly associated with the preference of home birth.

In this study, in Arba Minch demographic health surveillance site, the prevalence of preference of pregnant women to give birth at their home was 24% [95%CI: (19.9%-28.2%)]. World health organization and Ethiopia federal ministry of health encourage as every women give birth at health institution. However, this study showed that one fourth of pregnant women in this study area preferred home for place of delivery. This study is consistent with the research conducted in Wanago district Gedio, Ethiopia [12] which was 25.6%. Similarly, the finding of this study was in line with the study from Tanzania [9], which was 25.5%.

Homebirth preference in this study is higher than the research conducted in the Benchmaji zone, Ethiopia which was 12.1% [21]. The discrepancy could be due to the difference in the study participants, the preceding study was conducted among all married women’s where ours was among pregnant mothers. Similarly, the finding of this study was higher than the study in Debre Markos town, Ethiopia which was 19.6% [13]. This might be due to the difference in the study setting and study participants; the previous study was conducted in the town, and the study participants were pregnant women in second and third trimesters. Women who are living in urban have the chance of getting health access easily. In addition to this, they have a higher chance to get health-related information from different mass media than rural. Pregnant women’s preference for home birth decrease as gestational age increases because most pregnant women start ANC follow-up after the second trimester therefore they get advice and counseling about the benefit of institutional delivery.

The preference of home birth in this study was lower than the studies conducted in Ethiopia at Jimma Town [22], Debre Tabor town [19], Simada Amhara region [11], Sheshemenne [23], and South Tigrai zone which were 35.38%,29.2%,56.4%, 62.3%, and 28.8% respectively. The discrepancy might be due to the time difference, the development of health extension programs in training the HEWs, and the expansion of the health facilities in recent years. Furthermore, the study conducted in Simada Amhara region Ethiopia [11] was carried among women who gave birth. This might be due to the difference in the study participants. Because the last place of birth affects the current preferences, this is justified by the finding from a similar study shows that the choice of home birth in current pregnancy was comparable with the previous home birth [(home birth preference (56.4%) vs. Last home birth (56.6%)].

Husband involvement in decision making on birthplace was one factor associated with home birth preference. The odds of preference of home birth among pregnant women who decide birthplace with their husbands were 86% less likely compared to pregnant women who decide by themselves. This is supported by the study conducted in Awash Fantalle, Ethiopia [24]. This might be because most of the women in our country are dependent on their partners for decision-making and economics. Partner involvement through physical, emotional, and financial support from the perspective of maternal health service results in a positive outcome for utilization of health service [25].

The odds of preference of home birth among pregnant women who have no road access for transportation were 2.4 times higher compared to those who had road access for transportation. Similarly, the research was conducted in Simada, Ethiopia, [11], and Bangladesh [26]. Around 76 percent of Ethiopian women live in rural areas and do not have access to health care due to long traveling distances with lack of transportation [27]. Additionally, physiological changes during pregnancy like weight gain and easy fatigability may be challenges for a pregnant mother to travel a long distance to access health care.

Knowledge about obstetrical danger signs is significantly associated with home birth preference. Homebirth preference among participants who had poor knowledge about danger signs was 3 times higher compared to their counterparts. This is following the findings of studies conducted in Gura Dhamole Bale zone [28], Benishangul [29], Wonago District southern Ethiopia [12], and Ghana [30]. This might be due to knowledge about obstetrical danger signs from advice and counseling by a health professional, mass media, and other different sources helping pregnant women to increase their health-seeking behavior.

The odds of preference of home birth among respondents who did not hear about the benefit of institutional birth were 5.3 times higher compared to those who heard the benefit of institutional delivery. This is supported by the study conducted in Chencha Southern Ethiopia [18], and Uganda [31]. The possible reason could be that understanding the importance of giving birth in the institution helps pregnant women to prefer health institutions for delivery.

Another significant factor in this study was the attitude of study participants toward skilled birth services. The odds of preference for home birth among pregnant women who had negative attitudes toward skilled birth service were 3.1 times higher compared to the pregnant women who had a positive attitude. This is supported by the study conducted in Mizan Health Center, South West Ethiopia [32], and a study conducted in Benghazi, Libya [33]. Pieces of evidence showed that the health-seeking behavior of pregnant women is mainly affected by their attitude toward service given by health institutions [34].

The odds of preference for home birth among pregnant women who had high fear to give birth at health institutions were 5.1 times higher compared to their counterparts. This is supported by the studies conducted in two districts of West Gojjam Zone, Ethiopia [35], and Belgium and the Netherland [20]. This might be due to a lack of awareness about the care provided by health services. Childbirth fear is strongly linked to undesirable pregnancy outcomes and birth complications, such as prolonged labor, cesarean birth, birth traumas like fistulas, and weak emotional attachment in the postpartum period that affects maternal-infant interactions [36].

## Conclusions

In this study, in Arba Minch demographic health surveillance site, the prevalence of preference of pregnant women to give birth at their home was 24% [95%CI: (19.9%-28.2%)]. Husband involvement in decision making, no access of road for transportation, not heard about the benefit of institutional birth, poor knowledge about danger signs, negative attitude toward services, and high fear to give birth at health institutions were factors significantly associated with the preference of home birth.
