# Supplementary material for: Preference of homebirth and associated factors among pregnant women in Arba Minch health and demographic surveillance site, Southern Ethiopia
Source: PLoS One. 2022 Oct 27;17(10):e0276682. doi: 10.1371/journal.pone.0276682 (PMC9612484; doi:10.1371/journal.pone.0276682)
Supplement: Attachment — Submitted filename: Response to Reviewers.docx [file pone.0276682.s001.docx]

**RESPONSE TO REVIEWERS**

**Point raised**: Given the time period of the data collection

**Response**: from May 1 to June 1, 2021.

**Point raised:** the author's should address in the discussion how care-seeking or preference for home birth or the findings may have been affected by COVID-19 as well as the ongoing situation in Ethiopia, including conflict in other regions. This could be done by referring to existing data from other studies (i.e. past DHS, other qualitative research or survey conducted in that area of the country).

**Response**: COVID 19 was not their issue to prefer homebirth. The reason why COVID 19 was not their issue is that the study area is the surveillance site of Arba Minch University that the university gave the information about COVID 19 and the precaution should be applied during health care delivery for the community. As well as the community were immunized by the first round by Minister of health of Ethiopia since the community were living in the one of the five surveillance sites of the country. preliminary survey were conducted by the University after the above information and immunization were given for the community, indicated that community were free of the fear of the COVID 19 to seek health care because of they have been fully aware about the case and its precautions during health care-seeking and care-delivery. Thus, COVID 19 may not affect their preference.

**Response to the conflict in the country:** Southern region of the Ethiopia is one of the most stable regions in which there is no conflict still today and peaceful part of the country. Since the community was living in the area and seek health care from this the same area health facilities there is no chance to affect their preference of care.

**Point raised:** why data only available upon request?

**Response:**  The raw data documents are available upon request. Since the study area is one of the surveillance sites of the country it is not allowed to avail the data publically (legal restrictions on sharing). The data are only availed upon requested since it is restricted from public by Arba Minch University, college of medicine and health science due to data are owned by the organization. Therefore, data and other supplementary information can be obtained upon requested from surveillance site coordinator office of Arba Minch University, college of medicine, and health science ([ayelegistane@yahoo.com](mailto:ayelegistane@yahoo.com)).
