# Supplementary material for: Preference of homebirth and associated factors among pregnant women in Arba Minch health and demographic surveillance site, Southern Ethiopia
Source: PLoS One. 2022 Oct 27;17(10):e0276682. doi: 10.1371/journal.pone.0276682 (PMC9612484; doi:10.1371/journal.pone.0276682)
Supplement: Attachment — Submitted filename: RESPONSE TO REVIEWERS.docx [file pone.0276682.s004.docx]

**RESPONSE TO REVIEWERS # 1**

First of all we would like to express our deepest heartfelt thanks to the reviewers for your constructive comments, suggestions and questions to enrich our manuscript.

- **Point raised regarding to the title**
- **Responses**
- Preference of homebirth is the need of the pregnant mothers to give birth at their home. As they have intention to give birth at their home they may deliver at home which may lead to many life threatening complications. Thus why the title is mandatory to be research question for the identification of its prevalence and associated factors.
- In Ethiopian context, homebirth is not allowed for women instead going health facility for giving birth is the first priority. The reason is that home delivery is leading to many complications since it is not being conducted by skilled birth attendants but still now many pregnant mothers prefer to give birth at their home ignoring all life threatening complications. Thus, this research was aimed to identify prevalence and factors associated with this preference of homebirth.
- **Points raised in Abstract**
- **Responses**
- Missed opportunities changed to preferring homebirth because of the intention was talking about losing the care being given in health institutions since they preferred giving birth at home rather than health institutions.
- Study area Arba Minch zuria woreda surveillance site.
- The data were primary for this research and directly collected from the study participants.
- Negative attitude towards services and high fear to give birth at institution are two different independent variables which were independently assessed in multivariable binary logistic regression to identify association with preference of homebirth.
- Candidate variables in bi-variable binary logistic regression at p-value < 0.25 were transferred to multivariable binary logistic regression according to the rule of hosmer and lemishew as the model was fitted with hosmer and lemishew goodness of fit to identify the variables significantly associated with preference of homebirth.
- Measurements of preference of homebirth was obtained from the question asked to pregnant women; “where did you prefer to give birth?” Response to this question was either of home birth or health facility [hospital, health Centre/clinic, health post, and private hospital/clinic.
- The reason why such question is used for measurement of preference of place of birth preference in rural part of the Ethiopia starting from the traditional view to a now days left with the pregnant mother that they give birth at their preference area due to they are not flexible to the service being given at health institution thinking that traditional birth attendants give better services than health professionals. Additionally, this study was about the place preferred by pregnant mother for giving birth not about delivered birth place.
- Study participants were 416 pregnant mothers
- **Points raised about measurement of outcome variable (preference of home birth)**
- **Responses**
- The outcome variable “preference of homebirth” was obtained from the question asked to pregnant women; “where do you prefer to give birth?” Response to this question was either of home birth or health facility (hospital, health Centre/clinic, health post, and private hospital/clinic). Preference of homebirth is not equivalent with home delivery because it is only about the intention (preference) of the pregnant mothers want to give birth at their own home rather than preferring to deliver at health institutions. Therefore this outcome variable was only about the need of the pregnant mothers to give birth at their home.
- **Points raised regarding to justification of the study**
- **Responses**
- No study done on the preference of home birth among pregnant women in rural areas.  Additionally, this study addressed different variables (transportation facilities, benefits of institutional delivery, fear of child birth at institution) those were not addressed by previous studies.
- **Points raised regarding to assessment of knowledge and attitude**
- **Responses**
- **Knowledge about danger signs of pregnancy**, labor, and following childbirth: Knowledge about danger sign was assessed based on the women’s response to eight knowledge questions. Thus, women’s were considered as they have good knowledge if they answered correctly to four or more knowledge question.
- **Women’s Attitude about skilled birth services**: A total of 7 questions were used to assess attitude. Women responded to each question in the form of very agree, agree, disagree, and very disagree. Very agree and Agree was labeled as value "1", and disagree and very disagree was as assigned value "0". Women were considered as they have positive attitudes if all questions were labeled a value "1", and negative attitudes if any of the questions are labeled "0"
- **Points raised regarding to reference number of Ethical clearance**
- **Responses**
- An ethical clearance letter was obtained from Arba Minch University, college of medicine, and health sciences research ethics review board in 25/03/2021 with reference number IRB/1071/21.
- **Points raised regarding to conclusions:**
- The preference of home birth is 24% among pregnant women in the study area. Husband involvement in decision making, no access of road for transportation, not heard about the benefit of institutional birth, poor knowledge about danger signs, negative attitude toward services, and high fear to give birth at health institutions were factors significantly associated with the preference of home birth.

**RESPONSE TO REVIEWERS # 2**

First of all we would like to express our deepest heartfelt thanks to reviewer # 2 for your constructive comments, suggestions and questions to enrich our manuscript.

- **Points raised in Abstract**
- **Responses**
- Missed opportunities changed to preferring homebirth because of the intention was talking about losing the care being given in health institutions since they preferred giving birth at home rather than health institutions.
- Study area Arba Minch zuria woreda surveillance site.
- The data were primary for this research and directly collected from the study participants.
- Negative attitude towards services and high fear to give birth at institution are two different independent variables which were independently assessed in multivariable binary logistic regression to identify association with preference of homebirth.
- Candidate variables in bi-variable binary logistic regression at p-value < 0.25 were transferred to multivariable binary logistic regression according to the rule of hosmer and lemishew as the model was fitted with hosmer and lemishew goodness of fit to identify the variables significantly associated with preference of homebirth.
- Measurements of preference of homebirth was obtained from the question asked to pregnant women; “where did you prefer to give birth?” Response to this question was either of home birth or health facility [hospital, health Centre/clinic, health post, and private hospital/clinic.
- The reason why such question is used for measurement of preference of place of birth preference in rural part of the Ethiopia starting from the traditional view to a now days left with the pregnant mother that they give birth at their preference area due to they are not flexible to the service being given at health institution thinking that traditional birth attendants give better services than health professionals. Additionally, this study was about the place preferred by pregnant mother for giving birth not about delivered birth place.
- Study participants were 416 pregnant mothers
- **Points raised about measurement of outcome variable (preference of home birth)**
- **Responses**
- The outcome variable “preference of homebirth” was obtained from the question asked to pregnant women; “where do you prefer to give birth?” Response to this question was either of home birth or health facility (hospital, health Centre/clinic, health post, and private hospital/clinic). Preference of homebirth is not equivalent with home delivery because it is only about the intention (preference) of the pregnant mothers want to give birth at their own home rather than preferring to deliver at health institutions. Therefore this outcome variable was only about the need of the pregnant mothers to give birth at their home.
- **Measurements of variables**
- **Responses**
- **Knowledge about danger signs of pregnancy**, labor, and following childbirth: Knowledge about danger sign was assessed based on the women’s response to eight knowledge questions. Thus, women’s were considered as they have good knowledge if they answered correctly to four or more knowledge question.
- **Women’s Attitude about skilled birth services**: A total of 7 questions were used to assess attitude. Women responded to each question in the form of very agree, agree, disagree, and very disagree. Very agree and Agree was labeled as value "1", and disagree and very disagree was as assigned value "0". Women were considered as they have positive attitudes if all questions were labeled a value "1", and negative attitudes if any of the questions are labeled "0"
- **Points raised regarding to situation of Home birth Ethiopia**
- **Responses**
- According to mini Ethiopia public health 2019 report half of pregnant women in Ethiopia gave birth at home.
- All most all home delivery In Ethiopia attended without skilled birth attendants. In addition to this majority of Ethiopian population live in rural areas with lack of infrastructure including health sectors.
- Now a day facility delivery service provided by skilled birth attendants accredited from known university or college.
- **Points raised regarding to why study participant pregnant women is**
- **Responses**
- It is necessary to find out their needs and factors that affect care-seeking behavior in the given context. Because when they prefer to give birth at their home the probability of giving birth at institutions is very low since their internal need is not to delivery at health facilities, maternal mortality may follow the consequence.
- Women may give birth at home due to this; complications can occur during labor and delivery that is why we select the pregnant women as study participant. Then identifying the problem helps to intervene and create awareness that may come to their life due preference of homebirth.
- **Points raised regarding to techniques of sampling for this study**
- **Responses**
- Hence study was conducted in Arba Minch health and demographic surveillance site the list of pregnant women was obtained from health extension workers working in Arba Minch health and demographic surveillance. So the sample frame was obtained from the health extension workers in the surveillance site.
