# Supplementary material for: Preference of homebirth and associated factors among pregnant women in Arba Minch health and demographic surveillance site, Southern Ethiopia
Source: PLoS One. 2022 Oct 27;17(10):e0276682. doi: 10.1371/journal.pone.0276682 (PMC9612484; doi:10.1371/journal.pone.0276682)
Supplement: Attachment — Submitted filename: MANUSCRIPT OF HOME BIRTH PREFERENCE (1).docx [file pone.0276682.s005.docx]

PREFERENCE OF HOMEBIRTH AND ASSOCIATED FACTORS AMONG PREGNANT WOMEN IN ARBA MINCH HEALTH AND DEMOGRAPHIC SURVEILLANCE SITE, SOUTHERN ETHIOPIA

^1^ Solomon Seyife Alemu, ^2^Teklemariam Gultie Ketema, ^3^Kassahun Fikadu Tessema ^4*^Jira Wakoya Feyisa^, 5^Awol Arega Yimer, ^6^Birhanu Negese Kebede,

^1^ Mettu University College of health science department of midwifery

^2, 3, 5, 6^	Arba Minch University college of medicine and health science departments of Midwifery

^4^ Wollega University institute of health sciences department of public health, Nekemte, Ethiopia

Email addresses: [SoleSeifa@gmail.com](mailto:SoleSeifa@gmail.com) (S. Seyife), [teklemariam.gultie@amu.edu.et](mailto:teklemariam.gultie@amu.edu.et) (T. Gultie), [kasfika@gmail.com](mailto:kasfika@gmail.com) (K. Fikadu), [jirawakoya462@gmail.com](mailto:jirawakoya462@gmail.com) (J. Wakoya), [awolarega.aa@gmail.com](mailto:awolarega.aa@gmail.com) (A. Arega), [anbase103@gmail.com](mailto:anbase103@gmail.com) (B. Negese)

***Correspondent Author**: Jira Wakoya Feyisa, E-mail: [jirawakoya462@gmail.com](mailto:jirawakoya462@gmail.com), Phone: +251-923-00-95-83/+251-910-80-53-50

**ABSTRACT**

**Background**: Home birth preference is the need of pregnant women to give birth at their home with the help of traditional (unskilled) birth attendants. Homebirth with unskilled birth attendants during childbirth is the main leading indicator for maternal and newborn death. In most of developing countries like Ethiopia, home birth is assisted by unskilled personal. In Ethiopia, the numbers of women prefer homebirth. However, there is no information regarding the problem in the Arba Minch zuria woreda. Therefore, it is important to identify prevalence of preference of homebirth and associated factors.

**Objectives:** This study aimed to assess the preference of home birth and associated factors among pregnant women in Arba Minch health and demographic surveillance site, 2021.

**Method and materials**: A community-based cross-sectional study was conducted among 416 pregnant women in Arba Minch demographic health surveillance site, from May 1 to June 1, 2021. Study samples were selected through a simple random sampling technique. Data were collected through a face-to-face interview. Data were coded and entered into Epi-Data version 4.4.2.1 computer software and exported to Statistical Package for Social Sciences software version 25 for analysis. Bi-variable binary logistic regression for the selection of potential candidate variables at p-value < 0.25 for multivariable analysis and multivariable binary logistic regression to identify the association between homebirth preference and independent variables were carried out. The level of statistical significance was declared at a P-value < 0.05.

Result: The preference of home of birth was 24% [95%CI: (19.9%-28.2%)]. The factors significantly associated with the preference of home birth were husband involvement in decision making [AOR: 0.14 (0.05-0.38)], no access of road for transportation [AOR: 2.4 (1.2-5.18)], not heard about the benefit of institutional birth [AOR: 5.3 (2.3-12.2)], poor knowledge about danger signs [AOR: 3 (1.16-7.6)], negative attitude toward services [AOR: 3.1 (1.19-8.02)], and high fear to give birth at institution [AOR: 5.12 (2.4-10.91)].

**Conclusions:** The preference of home birth is 24% among pregnant women in the study area. Husband involvement in decision making, no access of road for transportation, not heard about the benefit of institutional birth, poor knowledge about danger signs, negative attitude toward services, and high fear to give birth at health institutions were factors significantly associated with the preference of home birth.

**Keywords:** Homebirth, Preference, Ethiopia

**INTRODUCTION**

According to the report in 2017, the coverage and progress of skilled birth attendants from the year 2012-2017 varied across the world, for instance, 54% in sub-Saharan countries versus 98% in Eastern Europe countries[1, 2]. In Ethiopia, the recent report shows that 52% of childbirths occurred at home with a lack of skilled birth attendants[3]. More than 50% of the risk of maternal mortality and 75% of stillbirth are reduced by providing emergency obstetrics care during labor and childbirth [4, 5]. However, in 2017 unevenly 295,000 women died due to pregnancy complications, labor and delivery and post natal complications more than 94% of this death occurred in low-income countries[6]. Ethiopia is one of the sub-Saharan countries with a high prevalence of maternal mortality ratio that accounts for 412 per 100,000 live births [7].

Different literature showed that women prefer home birth hence thinking childbirth at home is more comfortable, safer, and gives greater self-control than the health facilities[8, 9]. However, home birth in developing countries is attended by unskilled personal or family members with a lack of infrastructure[5]. Thus, home birth is difficult for early detection and management of complications like a failure of progress of labor, obstructed labor, postpartum hemorrhages, convulsion, infection, fetal distress, and others[8]. Even with highly trained health professionals, home birth is not safe in some conditions like heart disease, renal disease, diabetes, preeclampsia, hemorrhage, prior cesarean section delivery, and active genital warts[10].

In most circumstances, women attend health institutions after trial of labor and birth at their home [9]. The common reasons for attending health facilities are complications like retained placenta, excessive vaginal bleeding, shock, third and fourth-degree tear, cervical tear, uterine rupture, and severe anemia with the need for blood transfusion. These complications increase morbidity and mortality of the women[8, 9]. In addition to this, it increases the burden on health facilities and health professionals to manage the complications with limited resources[10].

In Ethiopia, the effort taken to reduce maternal and neonatal death inclusive of; providing free maternal service including, labor and delivery, extending health extension workers, health post and referral system[10]. Lack of knowledge on danger sign, poor road access, lack of ANC follow-up, and low educational status contributes to the preference of home birth [11-13].

The global community experts plan to overcome the challenges faced in the millennium development goals to sustainable development. By 2030 they aimed to decrease maternal mortality to 70 per 100,000 and neonatal mortality to 12 per 1000. These new strategies considered the shortage of resources and skilled personnel[14].

Safe delivery service is one of the crucial maternity care issues for pregnant women. It is necessary to find out the factors that affect care-seeking behavior in a given context. The majority of pregnant women in developing countries do not decide on a place of birth before the onset of labor. Women may give birth at health institutions without their preference by shifting their plans due to complications that occur during labor and delivery. In our country, most of the previous studies were conducted on institutional birth utilizations, not on their preference. The studies conducted in Ethiopia at Jimma town southwest, South Tigrai zone, Debre Tabor, and Debre Markos showed that the numbers of urban pregnant women prefer home birth. However, as far as our knowledge is concerned there was no study done on the preference of home birth among pregnant women in rural areas. Additionally, this study addressed different variables (transportation facilities, benefits of institutional delivery, fear of child birth at institution) those were not addressed by previous studies.

**METHODS AND MATERIALS**

**Study area, study design, and study population, and data collection period**

A community-based cross-sectional study was conducted from May 1- June 1, 2021, in Arba Minch Health and Demographic Surveillance Site. Arba Minch Health and Demographic Surveillance Site are located in Arba Minch Zuria and Gacho Baba districts, Gamo Zone, Southern Ethiopia, 500 km to the South of Addis Ababa, the capital city of Ethiopia. Arba Minch Zuria district and Gacho Baba district had a total of 31 kebeles [smallest administrative units] and it is included under Arba Minch Zuria Demographic and Health Development Program (AM-DHDP). AM-DHDP is owned by Arba Minch University and it is one of the six public universities Health and Demographic Surveillance System (HDSS) in Ethiopia. The surveillance site consists of nine kebeles which were selected in the representation of 31 kebeles in the district. From them, 6 kebeles were found in Arba Minch zuria district, and the rest three were found in Gacho baba districts. Farming is the predominant occupation of residents in the districts. Based on the 2007 census projection, the districts had a total population of 164,529. The district has 7 health centers and 37 health posts[15]. Around 81.8% of women gave birth at home in Arba Minch Zuria district[16].

**Source population**

All pregnant women living in Arba Minch health and demographic surveillance site were source population for this study.

**Study population**

Pregnant women living in selected nine Kebeles of Arba Minch health and demographic surveillance site were study population for this study.

**Inclusion criteria**

Pregnant women living in Arba Minch health and demographic surveillance site included in the study.

**Exclusion criteria**

Pregnant women with severely illness as well as those who were in labor during data collection period were excluded

**Sample size determination and sampling technique**

The sample size was determined by using a single population proportion formula, by considering the following assumptions; taking a proportion of home birth preference conducted in Simada district Ahmara region, Ethiopia, 56.4% proportion, 95% confidence level and power 80 considering 10 % non-response rates [11].

n = [(Zα/2)2p (1-p)]/d2 =378 by adding 10 % non-response rate the final sample size = **416**

**Where;**

n = the desired sample size.

Zα/2 = Standard normal deviate of 1.96 which corresponds to 95% confidence level (z value at

Alpha= 0.05).

P= Proportion home birth (56.4%).

d= an absolute precision (margin of error0 which is 5%.

Hence study was conducted in Arba Minch health and demographic surveillance site, the list of pregnant women was obtained from health extension workers working in Arba Minch health and demographic surveillance. The total number of pregnant women obtained from health extension workers from nine Kebeles of Arba Minch health and demographic surveillance site were 610. Before the selection of study participants, proportions to size allocations to each Kebeles were done. From the list, the required sample size (416) was selected by simple random sampling using computer-generated numbers.

**Data collection procedure**

The data were collected by using semi-structured interviewer-administered questionnaires. The questionnaires contain questions about socio-demographic characteristics, service-related, obstetrical characteristics, knowledge on danger signs, attitude toward skilled birth services, and fear of childbirth at a health institution. These questionnaires were adapted and developed from published related literatures [11, 12, 17-20]. Nine Health and demographic surveillance site data collectors and three supervisors were used. The data were collected by face-to-face interviews with participants at their homes. Preference of homebirth was obtained from the question asked to pregnant women; “where do you prefer to give birth?” Response to this question was either of home birth or health facility [hospital, health Centre/clinic, health post, and private hospital/clinic] [11].

**Data Quality Control**

To assure the data quality the questionnaires were translated from English to Amharic and retranslated to English for a consistent and proper check. The pre-test was done on a sample of 21 pregnant women (5 % of sample size) in Mirab Abaya woreda southern part of Ethiopia. The internal consistency of the tool was assessed by a reliability test (Cronbach’s alpha). The values of Cronbach’s alpha were 0.743, 0.841, and 0.919 for knowledge, attitude, and fear of childbirth at institution questions respectively. Two days of training on data collection procedures, and the objectives of the study for data collectors and supervisors were provided. Collected data was checked for completeness on daily basis by data collectors and supervisors.

**Data Processing and Analysis**

The collected data were coded and entered into Epi-Data version 4.4.2.1 software and exported to SPSS statistical software version 25 for data cleaning and further analysis. Errors related to the inconsistency of data were checked and corrected during data cleaning. Descriptive statistical analyses such as simple frequencies, percentage, median and interquartile range were used to describe the characteristics of participants.

The binary logistic regression model was fitted to identify factors associated with preference of homebirth after checking assumptions. Multi co-linearity by co-linearity matrix among the independent variables was checked. Bi-variable logistic regression analysis was performed between preference of homebirth and each of the independent variables, in sequence. Variables having a p-value of <0.25 in bi-variable logistic regression were a potential candidate for multivariable logistic regression analysis to control confounders in regression models. Variables having a p-value of less than 0.05 in the multivariable logistic regression model were considered as statistically significant. The final model was fitted with Hosmer and Lemeshow (p-value = 0.966). The strength of association between the preference of homebirth and independent variables were reported by using the adjusted odds ratio (AOR) with 95% CI.

**Ethical Consideration**

An ethical clearance letter was obtained from Arba Minch University, college of medicine, and health sciences research review board in 25/03/2021 with reference number IRB/1071/21. Written Permission was sought from the Health and demographic surveillance site, Arba Minch zuria and Gacho Baba districts. Written consent was obtained from each study participant before data collection and the purpose of the study was explained to the respondents. To protect confidentiality names and personal identification were not included in questionnaires. During data collection at the end of each interview women who prefer home birth were advised about the risk of home delivery. The issue of worldwide COVID 19 preventive approaches like social distancing face masks and hand sanitizer was practiced during data collection.

**Operational definitions**

**Women’s fear of childbirth at health institution:** A total of 13 items were presented to assess fear of childbirth at the health institution. Women responded to their level of fear for each item by a 4-point Likert scale. The women were classified as high fear if they scored mean value and above, and low fear if they scored less than mean value to question assessing fear of childbirth at institutions [19].

**Knowledge about danger signs of pregnancy, labor, and following childbirth:** Knowledge about danger sign was assessed based on the women’s response to eight knowledge questions. Thus, women’s were considered as they have good knowledge if they answered correctly to four or more knowledge question [20].

**Women’s Attitude about skilled birth services:** A total of 7 questions were used to assess attitude. Women responded to each question in the form of very agree, agree, disagree, and very disagree. Very agree and Agree was labeled as value "1", and disagree and very disagree was as assigned value "0". Women were considered as they have positive attitudes if all questions were labeled a value "1", and negative attitudes if any of the questions are labeled "0" [17]

# **RESULT****S**

**Socio-demographic characteristics of the study participants**

In this study, four hundred eight pregnant women volunteered to give information making a response rate of 98%. The median ages of the respondents were 29 years [interquartile range [(IQR) = (24-34)]. Regarding educational status, 165(40.4%) of the study participants and 173(45.3%) of respondents’ husbands were unable to read and write. Concerning occupational status, 244(59.8%) of study participants were housewives, and 156(40.8%) of their partners were a farmer. Eighty-eight point five percent of the participants were living in rural Kebeles. In terms of monthly household income, approximately half 188(46.1%) of the respondents earn <1000birr per month (Table 1).

**Table 1. Socio-demographic characteristics of the study participants in Arba Minch health demographic surveillance site in May 2021**

| **Variables** | Category | **Frequency** | **Percent [%]** |
| --- | --- | --- | --- |
| Age women in years categories | 18-19 | 46 | 11.3 |
|  | 20-24 | 69 | 16.9 |
|  | 25-29 | 107 | 26.2 |
|  | >=30 | 186 | 45.6 |
| Marital status | Married | 382 | 93.6 |
|  | Single | 19 | 4.7 |
|  | Separated | 7 | 1.7 |
| Ethnicity | Gamo | 312 | 76.5 |
|  | Gofa | 31 | 7.6 |
|  | Walayita | 52 | 12.7 |
|  | Gurage | 11 | 2.7 |
|  | Others * | 2 | 0.5 |
| Religion | Protestant | 198 | 48.5 |
|  | Muslim | 19 | 4.7 |
|  | Orthodox | 183 | 44.8 |
|  | Others ** | 8 | 2 |
| Women educational status | Unable to read and write | 165 | 40.4 |
|  | Primary education | 121 | 29.7 |
|  | Secondary education | 80 | 19.6 |
|  | Diploma and above | 42 | 10.3 |
| Women occupation | Housewife | 244 | 59.8 |
|  | Government employee | 33 | 8.1 |
|  | Merchant | 40 | 9.8 |
|  | Private employee | 39 | 9.6 |
|  | Student | 29 | 7.1 |
|  | Daily labor | 12 | 2.9 |
|  | Others*** | 11 | 2.7 |
| Husband educational status | Unable to read and write | 173 | 45.3 |
|  | Primary education | 79 | 20.7 |
|  | Secondary education | 77 | 20.2 |
|  | Diploma and above | 53 | 13.8 |
| Husband occupation | Government employee | 44 | 11.6 |
|  | Merchant | 62 | 16.2 |
|  | Farmer | 156 | 40.8 |
|  | Daily labor | 52 | 13.6 |
|  | Private employee | 54 | 14.1 |
|  | Student | 11 | 2.9 |
|  | Others*** | 3 | 0.8 |
| Residence | Rural | 361 | 88.5 |
|  | Urban | 47 | 11.5 |
| Number of household members | 1-5 | 311 | 76.2 |
|  | Above 5 | 97 | 23.8 |
| Household monthly income[ETB] | <1000 | 188 | 46.1 |
|  | 1001-2000 | 98 | 24 |
|  | >=2001 | 122 | 29.9 |

**Social and service-related characteristics**

In this study, more than half of pregnant women 225 (55.1%) decided place of birth with their husbands. Regarding the accessibility of maternal health services, 353(86.5%) of pregnant women had reported the distance of health facilities from their residences was less than 5km (Table 2**).**

Table 2. Social and service-related characteristics of study participants in Arba Minch health demographic surveillance site, May 2021.

| **Variables** | **Category** | **Frequency** | **Percent [%]** |
| --- | --- | --- | --- |
| Decision on the choice of place of birth | Women herself only | 104 | 25.5 |
|  | Both women and her husband | 225 | 55.1 |
|  | Only her husband | 59 | 14.7 |
|  | Traditional birth attendants | 3 | 0.5 |
|  | Her mother | 17 | 4.2 |
| Accessibilities of the road for transportation to ward health institution | Yes | 254 | 62.3 |
|  | No | 154 | 37.7 |
| Estimated distance from Health institution [km] | <=5km | 353 | 86.5 |
|  | >5km | 55 | 13.5 |

**Obstetric characteristics of the study respondents**

From obstetrical characteristics of respondents, almost near to three-fourth of respondents were multigravidas. One hundred eighty-nine participants gave birth to their last child at health institutions. Regarding the current pregnancy, 72.3% of respondents reported that their current pregnancy was wanted, and 69.1% of pregnant women have antenatal care follow-up for the current pregnancy. Sixty percent reported that they heard information about the benefits of institutional delivery and 28.7% of respondents reported at least one dangerous symptom during the current pregnancy (Table 3).

Table 3. Obstetrical characteristics of study participants in Arba Minch health demographic surveillance site, May 2021

| **Variables** | | **Category** | **Frequency** | **Percent** |
| --- | --- | --- | --- | --- |
| Gravida | 1 | | 114 | 27.9 |
|  | 2-5 | | 242 | 59.3 |
|  | >5 | | 52 | 12.8 |
| The interval between this pregnancy and the last pregnancy | <=1 year | | 15 | 5.1 |
|  | 2 -4years | | 218 | 74.2 |
|  | >=5 years | | 61 | 20.7 |
| Last place of birth | Institution | | 189 | 64.3 |
|  | Home | | 105 | 35.7 |
| Mode of delivery in last birth | SVD | | 240 | 81.6 |
|  | Assisted | | 30 | 10.2 |
|  | CS | | 24 | 8.2 |
| Maternal complications in last childbirth | Yes | | 57 | 19.4 |
|  | No | | 237 | 80.6 |
| The desire of this pregnancy | Wanted | | 295 | 72.3 |
|  | Unwanted | | 113 | 27.7 |
| Gestational age of this pregnancy [month] | <= 3months | | 25 | 6.1 |
|  | 4-6 months | | 102 | 25 |
|  | >=7 months | | 281 | 68.9 |
| ANC follow up for this pregnancy | Yes | | 282 | 69.1 |
|  | No | | 126 | 30.9 |
| Number of ANC visits | Once –Three | | 190 | 67.4 |
|  | Four and above | | 92 | 32.6 |
| Ever advised on the benefit of institutional birth during ANC | Yes | | 234 | 83 |
|  | No | | 48 | 17 |
| Heard about the benefit of institutional | Yes | | 245 | 60 |
|  | No | | 163 | 40 |
| Danger signs on this pregnancy | Yes | | 117 | 28.7 |
|  | No | | 291 | 71.3 |

**Pregnant women personal related characteristics**

**Knowledge about danger signs of pregnancy, labor, and following childbirth**

Concerning the knowledge about danger signs, 201(49.3%) of the participants had good knowledge about danger signs. Severe headaches, absent of fetal movement, and loss of consciousness were the danger signs mainly reported by the participants (Figure 1).

Knowledge of respondents

Frequency

| Danger sign on pregnancy, labor and folowing child birth | Frequency | Percent |
| --- | --- | --- |
| Absent of fetal movement | 80 | 19.5% |
| Convulsion | 76 | 14.2% |
| Blurred vision | 58 | 16.9% |
| Severe headache | 109 | 26.7% |
| Edema on upper extrimities | 59 | 14.5% |
| Excessive vaginal bleeding | 20 | 4.9% |
| Prolonged labor | 8 | 2% |
| Retained placenta | 5 | 1.2% |

Figure 1. Distribution of knowledge on danger signs of pregnancy, labor, and following childbirth among pregnant women in Arba Minch health demographic surveillance site, 2021.

**Attitude toward skilled care services and fear of childbirth at the institution**

Among respondents, 343(84.1%) pregnant women had a positive attitude toward skilled birth services. Regarding fear of childbirth at institutions, 229(56.1%) of the study participants had less fear of childbirth at health institutions.

**Pregnant mothers place of birth preference**

In this study 24% [95%CI: (19.9%, 28.2%)] of pregnant women were prefer home birth (Figure 2).

|  | Sales |
| --- | --- |
| Inistitutional | 76% |
| Home | 24% |

Figure 2. Distribution of pregnant women place birth preference in Arba Minch health demographic surveillance site May 2021

**Factors associated with home birth preference**

In this study, 10 variables were candidate for multivariable analysis (table 4). After controlling for potential confounders six variables; decision making on birthplace, road access for transportation to health institution, benefit of institutional delivery, knowledge about danger signs, the attitude toward skilled birth services, and fear of childbirth at health institution significantly associated with the preference of home delivery.

The odds of preference of home birth among pregnant women who decided birthplace with their husbands were 86% [AOR: 0.14 (0.05-0.38)] less likely compared to pregnant women who decided alone. Similarly, the odds of preference of home birth among pregnant women who have no road access for transportation were 2.4 times [AOR: 2.4 (1.2-5.18)] higher compared to those who have road access for transportation. The benefit of institutional birth was one of the factors significantly associated with the preference of home birth. The odds of preference of home birth among pregnant women who did not hear about the benefit of institutional birth were 5.3 times [AOR: 5.3 (2.3-12.2)] higher compared to those who heard about the benefit of institutional delivery. Knowledge about danger signs was significantly associated with the preference of home birth. The odds of preference of home of birth among pregnant women who had poor knowledge of danger signs were 3 times [AOR: 3 (1.16-7.6)] higher compared to pregnant women who had good knowledge on danger signs.

Attitude towards skilled birth services is also the other factor that is significantly associated with the preference of home birth. The odds of preference of home birth among pregnant women who had a negative attitude toward the skilled birth services were 3.1 times [AOR: 3.1 (1.19-8.02)], higher when compared with pregnant women who had a positive attitude toward the skilled birth services. The other significant variable was fear of childbirth at health institutions. The odds of preference of home birth among pregnant women who had high fear of childbirth at health institutions were 5.1 times [AOR: 5.12 (2.4-10.91)] higher compared to pregnant women who had less fear of childbirth at health institutions (table 4).

Table 4. Bivariable and multivariable logistic regression analysis of the home birth preference among pregnant women in Arba Minch Health demographic and surveillance site May 2021

| **Variables** | **Category** | **Place birth preference** | | **Crude odds ratio [95% CI]** | **Adjusted odds ratio 95%CI]** | **P-Value** |
| --- | --- | --- | --- | --- | --- | --- |
|  |  | **Home[98]** | **HI[310]** |  |  |  |
| Age of the women | 18-19 | 9[9.2%] | 37[11.9%] | 0.63[0.28-1.39] | 0.48[0.14-1.7] | 0.245 |
|  | 20-24 | 11[11.2%] | 58[18.7%] | 0.49[0.24-0.97] | 0.64[0.2-1.92] | 0.423 |
|  | 25-29 | 26[26.5%] | 81[26.1%] | 0.83[0.48-1.43] | 1.3[0.54-3.12] | 0.556 |
|  | >=30 | 52[53.1%] | 134[43.3%] | 1 |  |  |
| Educational status of women | Unable read and write | 62[63.3%] | 103[33.2%] | 5.52 [2.8-10.83] | 1.58[0.58-4.37] | 0.375 |
|  | Primary | 24[24.5%] | 97[31.3%] | 2.27[1.08-4.78] | 1.12[0.36-3.50] | 0.845 |
|  | Secondary and above | 12[12.2%] | 110[35.5%] | 1 | 1 |  |
| Marital status | In union | 80[81.6%] | 302[97.4%] | 1 | 1 |  |
|  | Not in union | 18[18.4%] | 8[2.6%] | 8.49[3.56-20.24] | 2.34 [0.7-7.84] | 0.168 |
| Income of household per month[Birr] | <=1000 | 60[61.2%] | 128[41.3%] | 4.730[2.37-9.44] | 1.7[0.59-4.93] | 0.326 |
|  | 1000-1999 | 27[27.6%] | 71[22.9%] | 3.84[1.79-8.22] | 1.77[0.5-6.03] | 0.358 |
|  | >=2000 | 11[11.2%] | 111[35.8%] | 1 | 1 |  |
| Road access for transportation to reach health institution | Yes | 35[35.7%] | 219[70.6%] | 1 | 1 |  |
|  | No | 63[64.3%] | 91[29.4%] | 4.33[2.68-7] | 2.4[1.2-5.18] | 0.024* |
| Decision making on the place of birth | Women her self | 38[38.7%] | 66[21.3%] | 1 |  |  |
|  | Both her and her husband | 13[13.3%] | 212[68.4%] | 0.11[0.05-0.21] | 0.14[0.05-0.38] | 0.001* |
|  | Husband, TBA, and her mother | 47[48%] | 32[10.3%] | 2.55[1.4-4.65] | 1.55[0.64-3.74] | 0.329 |
| Heard about the benefit of institutional delivery | Yes | 16[16.3] | 229[73.9%] | 1 | 1 |  |
|  | No | 82[83.7%] | 81[26.1%] | 14.5[8.01-26.21] | 5.3[2.3-12.2] | 0002* |
| Knowledge of women’s on danger sign | Good knowledge | 10[10.2%] | 191[61.6%] | 1 | 1 |  |
|  | Poor knowledge | 88[89.8%] | 119[38.4%] | 14.12[7.06-28.24] | 3[1.16-7.6] | 0.024* |
| The attitude of the respondents | Positive attitude | 63[35.7%] | 280[9.7%] | 1 | 1 |  |
|  | Negative attitude | 35[64.3%] | 30 [90.3%] | 5.19[2.97-9.07] | 3.1[1.19-8.02] | 0.020* |
| Fear of childbirth at the institution | High fear | 79[80.6%] | 100[32.3%] | 8.73[5.02-15.20] | 5.12[2.4-10.91] | 0.001* |
|  | Less fear | 19[19.4%] | 210[67.7%] | 1 | 1 |  |

# **DISCUSSION**

This study assessed the preference of home birth and associated factors among pregnant women in Arba Minch Health and demographic surveillance site, southern Ethiopia, 2021. Decision-making on birthplace, road accessibilities to health institutions, benefit of institutional delivery, knowledge about danger signs, the attitude toward skilled birth services, and fear of childbirth at health institutions were significantly associated with the preference of home birth.

In this study, the preference of home birth was **24% [95%CI: (19.9%-28.2%)]**. World health organization and Ethiopia federal ministry of health encourage as every women give birth at health institution. However, this study showed that one fourth of pregnant women in this study area preferred home for place of delivery. This study is consistent with the research conducted in Wanago district Gedio, Ethiopia[12] which was 25.6%. Similarly, the finding of this study was in line with the study from Tanzania[9], which was 25.5%.

Homebirth preference in this study is higher than the research conducted in the Benchmaji zone, Ethiopia which was 12.1% [21]. The discrepancy could be due to the difference in the study participants, the preceding study was conducted among all married women’s where ours was among pregnant mothers. Similarly, the finding of this study was higher than the study in Debre Markos town, Ethiopia which was 19.6%[13]. This might be due to the difference in the study setting and study participants; the previous study was conducted in the town, and the study participants were pregnant women in second and third trimesters. Women who are living in urban have the chance of getting health access easily. In addition to this, they have a higher chance to get health-related information from different mass media than rural. Pregnant women's preference for home birth decrease as gestational age increases because most pregnant women start ANC follow-up after the second trimester therefore they get advice and counseling about the benefit of institutional delivery.

The preference of home birth in this study was lower than the studies conducted in Ethiopia at Jimma Town[22], Debre Tabor town[19], Simada Amhara region[11], Sheshemenne [23], and South Tigrai zone which were 35.38%,29.2%,56.4%, 62.3%, and 28.8% respectively. The discrepancy might be due to the time difference, the development of health extension programs in training the HEWs, and the expansion of the health facilities in recent years.

Furthermore, the study conducted in Simada Amhara region Ethiopia[11] was carried among women who gave birth. This might be due to the difference in the study participants. Because the last place of birth affects the current preferences, this is justified by the finding from a similar study shows that the choice of home birth in current pregnancy was comparable with the previous home birth [(home birth preference (56.4%) vs. Last home birth (56.6%)].

Husband involvement in decision making on birthplace was one factor associated with home birth preference. The odds of preference of home birth among pregnant women who decide birthplace with their husbands were 86% less likely compared to pregnant women who decide by themselves. This is supported by the study conducted in Awash Fantalle, Ethiopia [24]. This might be because most of the women in our country are dependent on their partners for decision-making and economics. Partner involvement through physical, emotional, and financial support from the perspective of maternal health service results in a positive outcome for utilization of health service[25].

The odds of preference of home birth among pregnant women who have no road access for transportation were 2.4 times higher compared to those who had road access for transportation. Similarly, the research was conducted in Simada, Ethiopia, [11], and Bangladesh[26]. Around [76 percent](http://ethiopiahealth.blogs.wm.edu/ethiopian-health-system/) of Ethiopian women live in rural areas and do not have access to health care due to long traveling distances with lack of transportation[27]. Additionally, physiological changes during pregnancy like weight gain and easy fatigability may be challenges for a pregnant mother to travel a long distance to access health care.

Knowledge about obstetrical danger signs is significantly associated with home birth preference. Homebirth preference among participants who had poor knowledge about danger signs was 3 times higher compared to their counterparts. This is following the findings of studies conducted in Gura Dhamole Bale zone[28], Benishangul[29], Wonago District southern Ethiopia[12], and Ghana [30]. This might be due to knowledge about obstetrical danger signs from advice and counseling by a health professional, mass media, and other different sources helping pregnant women to increase their health-seeking behavior.

The odds of preference of home birth among respondents who did not hear about the benefit of institutional birth were 5.3 times higher compared to those who heard the benefit of institutional delivery. This is supported by the study conducted in Chencha Southern Ethiopia [18], and Uganda [31]. The possible reason could be that understanding the importance of giving birth in the institution helps pregnant women to prefer health institutions for delivery.

Another significant factor in this study was the attitude of study participants toward skilled birth services. The odds of preference for home birth among pregnant women who had negative attitudes toward skilled birth service were 3.1 times higher compared to the pregnant women who had a positive attitude. This is supported by the study conducted in Mizan Health Center, South West Ethiopia[32], and a study conducted in Benghazi, Libya [33]. Pieces of evidence showed that the health-seeking behavior of pregnant women is mainly affected by their attitude toward service given by health institutions[34].

The odds of preference for home birth among pregnant women who had high fear to give birth at health institutions were 5.1 times higher compared to their counterparts. This is supported by the studies conducted in two districts of West Gojjam Zone, Ethiopia[35], and Belgium and the Netherland[20]. This might be due to a lack of awareness about the care provided by health services. Childbirth fear is strongly linked to undesirable pregnancy outcomes and birth complications, such as prolonged labor, cesarean birth, birth traumas like fistulas, and weak emotional attachment in the postpartum period that affects maternal-infant interactions[36].

**Conclusions**

The preference of home birth is 24% among pregnant women in the study area. Husband involvement in decision making, no access of road for transportation, not heard about the benefit of institutional birth, poor knowledge about danger signs, negative attitude toward services, and high fear to give birth at health institutions were factors significantly associated with the preference of home birth.

**Acknowledgment:** We would like to acknowledge the Arba Minch zuria district administrator, Arba Minch health and demographic surveillance system coordinator, and data collectors who contributed to this work. We would like to thank all the participants for their participation and the information they provided us. We would like to extend our gratitude to Arba Minch University for all support and opportunity provided for us to conduct this study.

**Authors’ contribution:** SS, TG, KF, JW, AA, and BN conceived and designed the study, developed data collection instruments and supervised data collection. SS, JW, AA, and BN participated in the testing and finalization of the data collection instruments and coordinated the study progress. SS performed the statistical analysis and wrote all versions of the manuscript. All authors read and approved the final manuscript.

**Competing interests:** The authors declare that there was no competing interest

**Consent to publish:** Not applicable.

**Availability of data and materials:** The raw data documents are available upon request. Since the study area is one of the surveillance sites of the country it is not allowed to avail the data publically [legal restrictions on sharing]. The data are only availed upon requested since it is restricted from public by Arba Minch University, college of medicine and health science due to data are owned by the organization. Therefore, data and other supplementary information can be obtained upon requested from surveillance site coordinator office of Arba Minch University, college of medicine, and health science [[ayelegistane@yahoo.com](mailto:ayelegistane@yahoo.com)].

**Funding:** Not applicable

**REFERENCE**

1.	UNICEF/WHO. skilled health personnel, based on population based national household survey data and routine health systems. 2019.

2.	UNICEF. based on MICS, DHS and other nationally representative household surveys. global databases. 2021.

3.	Ethiopian Public Health Institute E, Federal Ministry of Health F, Icf. Ethiopia Mini Demographic and Health Survey. Ethiopian Public Health Institute [EPHI] [Ethiopia] and ICF. 2021.

4.	Ameh CA, Mdegela M, White S, van den Broek N. The effectiveness of training in emergency obstetric care: a systematic literature review. Health policy and planning. 2019;34[4]:257-70.

5.	Yakoob MY, Ali MA, Ali MU, Imdad A, Lawn JE, Van Den Broek N, et al. The effect of providing skilled birth attendance and emergency obstetric care in preventing stillbirths. BMC Public Health. 2011;11[3]:S7.

6.	UNFPA WHO, UNICEF, World Bank Group, the United Nations Population Division. : 2000 to 2017: estimates by WHO, UNICEF, UNFPA, World Bank Group and the United Nations Population Division. Geneva: . World Health Organization 2019;978-92-4-151648-8.

7.	Ethiopian Public Health Institute E, Federal Ministry of Health F, Icf. Ethiopia Demographic and Health Survey 2016. Addis Ababa, Ethiopia, and Rockville, Maryland, USA:. CSA and ICF 2016.

8.	Tuladhar.H. complication of home delivery: our exprience at Nepal Medical College Teaching Hospital. JNMA J Nepal Med Assoc. 2010;44[159][87-91].

9.	Exavery A K, Njozi M, Tani K, Doctor HV, Hingora A, Phillips Access to institutional delivery care and reasons for home delivery in three districts of Tanzania. International journal for equity in health 2014;13[1]:48.

10.	Karlsen S, Say L, Souza J-P, Hogue CJ, Calles DL, Gülmezoglu AM, et al. The relationship between maternal education and mortality among women giving birth in health care institutions: Analysis of the cross sectional WHO Global Survey on Maternal and Perinatal Health. BMC Public Health. 2011;11[1]:606.

11.	Mekie M, Taklual W. Delivery place preference and its associated factors among women who deliver in the last 12 months in Simada district of Amhara Region, Northwest Ethiopia: a community based cross sectional study. BMC Research Notes. 2019;12[1]:114.

12.	Alemu A. Women’s Preference of Home Delivery in Wonago District, Gedeo Zone, Southern Ethiopia 2018. Journal of Gynecology and Obstetrics. 2019;7:85-91.

13.	Bayu H, Adefris M, Amano A, Abuhay M. Pregnant women’s preference and factors associated with institutional delivery serviceutilization in Debra Markos Town, North WestEthiopia: a community based follow up study. BMC Pregnancy and Childbirth. 2015;15[15].

14.	World: NUGATo. The 2030 agenda for sustainable development; . United Nations Statistics Division [UNSD] 2015;A/RES/70/1.

15.	Arbaminch, office ZHD. Arbaminch zuria district health profiles. 2021.

16.	Ayele G, Tilahun M, Merdekios B, Animaw W, Taye W. Prevalence and associated factors of home delivery in Arbaminch Zuria district, southern Ethiopia: Community based cross sectional study. Science Journal of Public Health. 2015;3:6-9.

17.	Bayu H, et, al. Missed opportunities for institutional delivery and associated factors among urban resident pregnant women in South Tigray Zone, Ethiopia: a community-based follow-up study. Glob Health Action. 2015;8[28082].

18.	Mensa M. Status of Skilled Birth Attendance Utilization and Determinants Among Women of Child Bearing Age in Chencha Woreda, Gamo Gofa Zone, Southern Ethiopia, December 2016. . JOJ Case Stud. 2017;4[3].

19.	Tsegaye B, Abuhay M, Admasu E, Wubale B, Temesgen K, Yohannes Z. Level and factors associated with preference of institutional delivery among pregnant woman in Debre-tabor town, North West Ethiopia, 2017: a community based cross sectional study. BMC research note. 2019;12[44].

20.	Christiaens W, Van De Velde S, Bracke P. Pregnant women's fear of childbirth in midwife- and obstetrician-led care in Belgium and the Netherlands: test of the medicalization hypothesis. Women Health. 2011;51[3]:220-39.

21.	Ababulgu FA, Bekuma TT. Delivery site preferences and associated factors among married women of child bearing age in Bench Maji Zone, Ethiopia. Ethiop J Health Sci. 2016;26[1]:45-54.

22.	Tsegaye R, Belina S. Assessment of Factors Affecting Choice of Delivery Place among Pregnant Women in Jimma Zone, South West Ethiopia: Cross Sectional Study. Women’s Health Care. 2014;4:1.

23.	Junayde Abdurahmen AA, Mitike Molla. Community Based Cross-Sectional Study:Preferences Of Placeofdelivery And BirthAttendants Among Women Of shashemeneTown, Oromia Regional State International journal of technology enhancements and emerging engineering research,. 2012;2[7]:2347-4289.

24.	Assefa L, Alemayehu M, Debie A. Magnitude of institutional delivery service utilization and associated factors among women in pastoral community of Awash Fentale district Afar Regional State, Ethiopia. BMC Research Notes. 2018;11[1]:162.

25.	Tessema KM, Mihirete KM, Mengesha EW, Nigussie AA, Wondie AG. The association between male involvement in institutional delivery and women’s use of institutional delivery in Debre Tabor town, North West Ethiopia: Community based survey. PLOS ONE. 2021;16[4]:e0249917.

26.	Sarker BK, Rahman M, Rahman T, Hossain J, Reichenbach L, Mitra DK. Reasons for Preference of Home Delivery with Traditional Birth Attendants [TBAs] in Rural Bangladesh: A Qualitative Exploration. PLoS One. 2016;11[1]:e0146161.

27.	Hibbett K. <https://borgenproject.org/addressing-the-barriers-to-proper-health-care-in-ethiopia/#top> 2018.

28.	Ayele, Et, al. Utilization of skilled birth attendant at birth and associated factors among women who gave birth in the last 24months preceding the survey in Gura Dhamole Woreda, Bale zone, southeast Ethiopia BMC Public Health. 2019;19:1505.

29.	Weldemariam S, Kiros A, Welday M. Utilization of institutional delivery service and associated factors among mothers in North West Ethiopian. BMC Research Notes. 2018;11[1]:194.

30.	Nakua EK, Sevugu JT, Dzomeku VM, Otupiri E, Lipkovich HR, Owusu-Dabo E. Home birth without skilled attendants despitemillennium villages project intervention in Ghana: insight from a survey of women’s perceptions of skilled obstetric care. BMC Pregnancy and Childbirth 2015;15:243.

31.	Anastasi, et, al. Losing women along the path to safe motherhood: why is there such a gap between women’s use of antenatal care and skilled birth attendance? A mixed methods study in northern Uganda BMC Pregnancy and Childbirth. 2015;15:287.

32.	Henok A, Worku H, Getachew H, Workiye H. Knowledge, Attitude and Practice of Antenatal Care Service among Married Women of Reproductive Age Group in Mizan Health Center, South West Ethiopia. Journal of Medicine, Physiology and Biophysics. 2015;16.

33.	Kadry H, Borgy M, Mohammed H. Knowledge, attitude, and practices of pregnant women towards antenatal care in primary healthcare centers in Benghazi, Libya. The Journal of the Egyptian Public Health Association. 2014;89:119-26.

34.	Peprah P, Mawuli Abalo E, Nyonyo J, Okwei R, Agyemang-Duah W, Amankwaa G. Pregnant women’s perception and attitudes toward modern and traditional midwives and the perceptional impact on health seeking behaviour and status in rural Ghana. International Journal of Africa Nursing Sciences. 2018;8:66-74.

35.	Shiferaw BB, Modiba LM. Why do women not use skilled birth attendance service? An explorative qualitative study in north West Ethiopia. BMC Pregnancy and Childbirth. 2020;20[1]:633.

36.	Adams SS, Eberhard-Gran M, Eskild A. Fear of childbirth and duration of labour: a study of 2206 women with intended vaginal delivery. BJOG : an international journal of obstetrics and gynaecology. 2012;119[10]:1238-46.
