# Supplementary material for: Preference of homebirth and associated factors among pregnant women in Arba Minch health and demographic surveillance site, Southern Ethiopia
Source: PLoS One. 2022 Oct 27;17(10):e0276682. doi: 10.1371/journal.pone.0276682 (PMC9612484; doi:10.1371/journal.pone.0276682)
Supplement: Attachment — Submitted filename: RESPONSE TO REVIEWERS.docx [file pone.0276682.s006.docx]

**RESPONSE TO EDITOR**

First of all we would like to express our deepest heartfelt thanks to the editor for your constructive comments, suggestions and questions to enrich our manuscript.

- **Point raised regarding to the article appears to be a secondary data analysis**
- **Responses**
- The study entitled with “Preference of homebirth and associated factors among pregnant women in Arba Minch health and demographic surveillance site, southern Ethiopia” was conducted by collecting primary data from selected pregnant women of Arba Minch health and demographic surveillance site. Therefore the title was the research of primary data
- The data were primary for this research and directly collected from the study participants.
- Preference of homebirth is the need of the pregnant mothers to give birth at their home. As they have intention to give birth at their home they may deliver at home which may lead to many life threatening complications. Thus why the title is mandatory to be research question for the identification of its prevalence and associated factors.
- In Ethiopian context, homebirth is not allowed for women instead going health facility for giving birth is the first priority. The reason is that home delivery is leading to many complications since it is not being conducted by skilled birth attendants but still now many pregnant mothers prefer to give birth at their home ignoring all life threatening complications. Thus, this research was aimed to identify prevalence and factors associated of preference of homebirth using primary data collected.
- **Points raised regarding to variables in regression**
- **Responses**
- **Study variables**
- **Dependent variable**
- Preference of home birth
- “Preference of home birth” was the dependent variable and was obtained from the question, “Where do you prefer/need to give birth [choices]?” Response to this question was prefer/need to give birth at home or at government hospital/health center or private hospital/clinic. It was then dichotomized to into prefer facility birth = 0 and prefer home birth = 1 where respondent’s preference/need to give birth at home “prefer home birth” and all the other categories were grouped as “prefer facility birth”.
- **Independent variables**
- The independent variables considered in this study were age of the women, marital status, ethnicity, religion, women educational status, women occupation, husband educational status, husband occupation, household income, residence, family size, Gravid, pregnancy desire, last place of delivery, last mode of delivery, last birth complication, current ANC status, number of ANC follow up, birth interval, distance from health services/facility, road access for transportation to health institutions, information on the benefit of institutional birth, Knowledge of danger signs, attitude toward skilled birth services, decision-making, and fear of childbirth at the institution
- Ten (10) candidate variables in bi-variable binary logistic regression at p-value < 0.25 were transferred to multivariable binary logistic regression according to the rule of hosmer and lemishew as the model was fitted with hosmer and lemishew goodness of fit to identify the variables significantly associated with preference of homebirth.
- **Points raised regarding to description of the methods specifically Study area, study design, and study population, data collection period and study variables to be described using separate sub-headings**
- **Response**
- Separately described in manuscript according to reviewer enquire of sub-headings separately.

**RESPONSE TO REVIEWER # 2**

First of all we would like to express our deepest heartfelt thanks to reviewer # 2 for your constructive comments, suggestions and questions to enrich our manuscript.

- **Point raised regarding to the conclusion to state what does the found 24% mean?**
- **Response**
- This indicates that in this study, in Arba Minch demographic health surveillance site, the prevalence of the need (preference) of pregnant women to give birth at their home was 24% [95%CI: (19.9%-28.2%)].
- **Point raised regarding to separate the subheadings so that a reader can follow, study design and setting stand together but other sub-headings such as sampling technique and others need their own sub-heading**
- **Responses**
- Separately done as per of the enquiry
- **Study design and study area**

A community-based cross-sectional study was conducted in Arba Minch Health and Demographic Surveillance Site. Arba Minch Health and Demographic Surveillance Site are located in Arba Minch Zuria and Gacho Baba districts, Gamo Zone, Southern Ethiopia, 500 km to the South of Addis Ababa, the capital city of Ethiopia. Arba Minch Zuria district and Gacho Baba district had a total of 31 kebeles [smallest administrative units] and it is included under Arba Minch Zuria Demographic and Health Development Program (AM-DHDP). AM-DHDP is owned by Arba Minch University and it is one of the six public universities Health and Demographic Surveillance System (HDSS) in Ethiopia. The surveillance site consists of nine kebeles which were selected in the representation of 31 kebeles in the district. From them, 6 kebeles were found in Arba Minch zuria district, and the rest three were found in Gacho baba districts. Farming is the predominant occupation of residents in the districts. Based on the 2007 census projection, the districts had a total population of 164,529. The district has 7 health centers and 37 health posts. Around 81.8% of women gave birth at home in Arba Minch Zuria district.

- **Data collection period**

Data were collected from May 1- June 1, 2021 from randomly selected pregnant women of Arba Minch zuria woreda.

- **Study population**

Pregnant women living in selected nine Kebeles of Arba Minch health and demographic surveillance site were study population for this study.

- **Points raised regarding to techniques of how was it possible for them to do random sampling in a community based study**
- **Responses**
- Hence study was conducted in Arba Minch health and demographic surveillance site registration and identification of the women becoming pregnant with their address is one of the core and continuum activities of the health extension workers assigned to the woreda. As well registration and identification of the women becoming pregnant with their address are common activity in all parts of Ethiopia. Thus lists of pregnant women were obtained from health extension workers working in Arba Minch health and demographic surveillance. So the sampling frames were obtained from the health extension workers in the surveillance site then simple random sampling technique was applied using computer random generated numbers.
- **Points raised regarding to Variable measurement section is needed in the method**
- **Response**
- **Modification was done as follows in the manuscript**
- **Study variables**
- **Dependent variable**
- Preference of home birth
- “Preference of home birth” was the dependent variable and was obtained from the question, “Where do you prefer/need to give birth [choices]?” Response to this question was prefer/need to give birth at home or at government hospital/health center or private hospital/clinic. It was then dichotomized to into prefer facility birth = 0 and prefer home birth = 1 where respondent’s preference/need to give birth at home “prefer home birth” and all the other categories were grouped as “prefer facility birth” [11].
- **Independent variables**
- The independent variables considered in this study were age of the women, marital status, ethnicity, religion, women educational status, women occupation, husband educational status, husband occupation, household income, residence, family size, Gravid, pregnancy desire, last place of delivery, last mode of delivery, last birth complication, current ANC status, number of ANC follow up, birth interval, distance from health services/facility, road access for transportation to health institutions, information on the benefit of institutional birth, Knowledge of danger signs, attitude toward skilled birth services, decision-making, and fear of childbirth at the institution
- **Operational definitions**
- **Women’s fear of childbirth at health institution:** A total of 13 items were presented to assess fear of childbirth at the health institution. Women responded to their level of fear for each item by a 4-point Likert scale. The women were classified as high fear if they scored mean value and above, and low fear if they scored less than mean value to question assessing fear of childbirth at institutions [19].
- **Knowledge about danger signs of pregnancy, labor, and following childbirth:** Knowledge about danger sign was assessed based on the women’s response to eight knowledge questions. Thus, women’s were considered as they have good knowledge if they answered correctly to four or more knowledge question [20].
- **Women’s Attitude about skilled birth services:** A total of 7 questions were used to assess attitude. Women responded to each question in the form of very agree, agree, disagree, and very disagree. Very agree and Agree was labeled as value "1", and disagree and very disagree was as assigned value "0". Women were considered as they have positive attitudes if all questions were labeled a value "1", and negative attitudes if any of the questions are labeled "0" [17]

- **Point raised regarding to discussion and language edition**
- **Response**
- Modification were made to enrich discussion part especially factor related part**.**
- “Was” changed to “were” following the word data**.**
