# Supplementary material for: Preference of homebirth and associated factors among pregnant women in Arba Minch health and demographic surveillance site, Southern Ethiopia
Source: PLoS One. 2022 Oct 27;17(10):e0276682. doi: 10.1371/journal.pone.0276682 (PMC9612484; doi:10.1371/journal.pone.0276682)
Supplement: Attachment — Submitted filename: RESPONSE TO REVIEWERS.docx [file pone.0276682.s007.docx]

**RESPONSE TO REVIEWER # 2**

First of all we would like to express our deepest heartfelt thanks to reviewer # 2 for your constructive comments, suggestions and questions to enrich our manuscript.

- **In the background information, include other factors associated with choices of place for childbirth as reported in the literature**
- **Response**
- As one of the components should be explained in the introduction part the common factors contributed to the preference of home birth from the literatures were age of the pregnant mothers, Lack of knowledge on danger sign, poor road access, lack of ANC follow-up, low household income, place of last delivery, parity, and low educational status (11-13).
- **Sampling technique is not well elaborated, how did you ensure probability sampling**
- **Responses**
- Simple random probability sampling technique was used in this study to ensure the representativeness of the information because lists of pregnant women is available as every pregnant mother in every district and Kebeles should be registered by health extension workers of Ethiopia. Thus why their data which contains these pregnant mothers is always available and researcher can get the lists and use for scientific purposes. Therefore, simple random sampling technique was ensured due to the lists/sampling frame of the pregnant mother of the study area were available.
- Hence study was conducted in Arba Minch health and demographic surveillance site registration and identification of the women becoming pregnant with their address is one of the core and continuum activities of the health extension workers assigned to the woreda. As well registration and identification of the women becoming pregnant with their address are common activity in all parts of Ethiopia. Thus lists of pregnant women were obtained from health extension workers working in Arba Minch health and demographic surveillance. So the sampling frames were obtained from the health extension workers in the surveillance site then simple random sampling technique was applied using computer random generated numbers.
- **In the data collection I suggest you change the use of face to face interview to interviewer administered questionnaire because mostly face to face interview is used in qualitative studies**
- **Responses**
- As per the recommendation face to face interview is changed to “interviewer-administered questionnaire” in the manuscript.
