# Supplementary material for: Preference of homebirth and associated factors among pregnant women in Arba Minch health and demographic surveillance site, Southern Ethiopia
Source: PLoS One. 2022 Oct 27;17(10):e0276682. doi: 10.1371/journal.pone.0276682 (PMC9612484; doi:10.1371/journal.pone.0276682)
Supplement: Peer review history

# Editor report: Decision Letter 0

**DOI:** 10.1371/journal.pone.0276682.r001

**Authors:** Anayda Portela

6 Jan 2022

PONE-D-21-39161PREFERENCE OF HOMEBIRTH AND ASSOCIATED FACTORS AMONG PREGNANT WOMEN IN ARBA MINCH HEALTH AND DEMOGRAPHIC SURVEILLANCE SITE, SOUTHERN ETHIOPIAPLOS ONE

Dear Dr. Feyisa,

Thank you for submitting your manuscript to PLOS ONE. After careful consideration, we feel that it has merit but does not fully meet PLOS ONE’s publication criteria as it currently stands. Therefore, we invite you to submit a revised version of the manuscript that addresses the points raised during the review process.

==============================

Prior to submitting for peer review, we would like to ask you to address the following. Given the time period of the data collection, the author's should address in the discussion how care-seeking or preference for home birth or the findings may have been affected by COVID-19 as well as the ongoing situation in Ethiopia, including conflict in other regions. This could be done by referring to existing data from other studies (i.e. past DHS, other qualitative research or survey conducted in that area of the country).

==============================

Please submit your revised manuscript by Feb 20 2022 11:59PM. If you will need more time than this to complete your revisions, please reply to this message or contact the journal office at plosone@plos.org. When you're ready to submit your revision, log on to https://www.editorialmanager.com/pone/ and select the 'Submissions Needing Revision' folder to locate your manuscript file.

Please include the following items when submitting your revised manuscript:

- A rebuttal letter that responds to each point raised by the academic editor and reviewer(s). You should upload this letter as a separate file labeled 'Response to Reviewers'.
- A marked-up copy of your manuscript that highlights changes made to the original version. You should upload this as a separate file labeled 'Revised Manuscript with Track Changes'.
- An unmarked version of your revised paper without tracked changes. You should upload this as a separate file labeled 'Manuscript'.

If you would like to make changes to your financial disclosure, please include your updated statement in your cover letter. Guidelines for resubmitting your figure files are available below the reviewer comments at the end of this letter.

If applicable, we recommend that you deposit your laboratory protocols in protocols.io to enhance the reproducibility of your results. Protocols.io assigns your protocol its own identifier (DOI) so that it can be cited independently in the future. For instructions see: https://journals.plos.org/plosone/s/submission-guidelines#loc-laboratory-protocols. Additionally, PLOS ONE offers an option for publishing peer-reviewed Lab Protocol articles, which describe protocols hosted on protocols.io. Read more information on sharing protocols at https://plos.org/protocols?utm_medium=editorial-email&utm_source=authorletters&utm_campaign=protocols.

We look forward to receiving your revised manuscript.

Kind regards,

Anayda Portela

Academic Editor

PLOS ONE

Journal Requirements:

When submitting your revision, we need you to address these additional requirements.

1. Please ensure that your manuscript meets PLOS ONE's style requirements, including those for file naming. The PLOS ONE style templates can be found at 

https://journals.plos.org/plosone/s/file?id=wjVg/PLOSOne_formatting_sample_main_body.pdf and 

https://journals.plos.org/plosone/s/file?id=ba62/PLOSOne_formatting_sample_title_authors_affiliations.pdf.

2. You indicated that you had ethical approval for your study. In your Methods section, please ensure you have also stated whether you obtained consent from parents or guardians of the minors included in the study or whether the research ethics committee or IRB specifically waived the need for their consent.

3. We note that you have indicated that data from this study are available upon request. PLOS only allows data to be available upon request if there are legal or ethical restrictions on sharing data publicly. For more information on unacceptable data access restrictions, please see http://journals.plos.org/plosone/s/data-availability#loc-unacceptable-data-access-restrictions. 

In your revised cover letter, please address the following prompts:

a) If there are ethical or legal restrictions on sharing a de-identified data set, please explain them in detail (e.g., data contain potentially sensitive information, data are owned by a third-party organization, etc.) and who has imposed them (e.g., an ethics committee). Please also provide contact information for a data access committee, ethics committee, or other institutional body to which data requests may be sent.

b) If there are no restrictions, please upload the minimal anonymized data set necessary to replicate your study findings as either Supporting Information files or to a stable, public repository and provide us with the relevant URLs, DOIs, or accession numbers. For a list of acceptable repositories, please see http://journals.plos.org/plosone/s/data-availability#loc-recommended-repositories.

We will update your Data Availability statement on your behalf to reflect the information you provide.

4. Your ethics statement should only appear in the Methods section of your manuscript. If your ethics statement is written in any section besides the Methods, please move it to the Methods section and delete it from any other section. Please ensure that your ethics statement is included in your manuscript, as the ethics statement entered into the online submission form will not be published alongside your manuscript.

5. Please review your reference list to ensure that it is complete and correct. If you have cited papers that have been retracted, please include the rationale for doing so in the manuscript text, or remove these references and replace them with relevant current references. Any changes to the reference list should be mentioned in the rebuttal letter that accompanies your revised manuscript. If you need to cite a retracted article, indicate the article’s retracted status in the References list and also include a citation and full reference for the retraction notice.

[Note: HTML markup is below. Please do not edit.]

Reviewers' comments:

[NOTE: If reviewer comments were submitted as an attachment file, they will be attached to this email and accessible via the submission site. Please log into your account, locate the manuscript record, and check for the action link "View Attachments". If this link does not appear, there are no attachment files.]

While revising your submission, please upload your figure files to the Preflight Analysis and Conversion Engine (PACE) digital diagnostic tool, https://pacev2.apexcovantage.com/. PACE helps ensure that figures meet PLOS requirements. To use PACE, you must first register as a user. Registration is free. Then, login and navigate to the UPLOAD tab, where you will find detailed instructions on how to use the tool. If you encounter any issues or have any questions when using PACE, please email PLOS at figures@plos.org. Please note that Supporting Information files do not need this step.

# Author comment: Author response to Decision Letter 0

**DOI:** 10.1371/journal.pone.0276682.r002

19 Jan 2022

RESPONSE TO REVIEWERS

Point raised: Given the time period of the data collection

Response: from May 1 to June 1, 2021.

Point raised: the author's should address in the discussion how care-seeking or preference for home birth or the findings may have been affected by COVID-19 as well as the ongoing situation in Ethiopia, including conflict in other regions. This could be done by referring to existing data from other studies (i.e. past DHS, other qualitative research or survey conducted in that area of the country).

Response: COVID 19 was not their issue to prefer homebirth. The reason why COVID 19 was not their issue is that the study area is the surveillance site of Arba Minch University that the university gave the information about COVID 19 and the precaution should be applied during health care delivery for the community. As well as the community were immunized by the first round by Minister of health of Ethiopia since the community were living in the one of the five surveillance sites of the country. preliminary survey were conducted by the University after the above information and immunization were given for the community, indicated that community were free of the fear of the COVID 19 to seek health care because of they have been fully aware about the case and its precautions during health care-seeking and care-delivery. Thus, COVID 19 may not affect their preference.

Response to the conflict in the country: Southern region of the Ethiopia is one of the most stable regions in which there is no conflict still today and peaceful part of the country. Since the community was living in the area and seek health care from this the same area health facilities there is no chance to affect their preference of care.

Point raised: why data only available upon request?

Response: Since the study area is one of the surveillance sites of the country it is not allowed to avail the data publically. The data are only availed upon requested since restriction was given us to avail the data for the one in need only upon request. Therefore data and other supplementary information can be obtained upon requested from Correspondent Author.

Attachment Submitted filename: Response to Reviewers.docx Click here for additional data file.

# Peer review: Decision Letter 1

**DOI:** 10.1371/journal.pone.0276682.r003

**Authors:** Anayda Portela

9 Mar 2022

PONE-D-21-39161R1PREFERENCE OF HOMEBIRTH AND ASSOCIATED FACTORS AMONG PREGNANT WOMEN IN ARBA MINCH HEALTH AND DEMOGRAPHIC SURVEILLANCE SITE, SOUTHERN ETHIOPIAPLOS ONE

Dear Dr. Feyisa,

Thank you for submitting your manuscript to PLOS ONE. After careful consideration, we feel that it has merit but does not fully meet PLOS ONE’s publication criteria as it currently stands. Therefore, we invite you to submit a revised version of the manuscript that addresses the points raised during the review process.  You will find attached comments from the reviewers as well as my own comments in an attachment.  We hope you find them helpful.

==============================

- There are several concerns regarding the methods and a description of the methods.  The article appears to be a secondary data analysis but this is not stated.  The study variables, particularly the outcome variable, are not clearly explained; the reviewers were confused as to the outcome used in your model. Only the variables that showed statistical significance in the bivariate analysis should be used for the regression analysis.
- I refer you to an article that may be useful to you as you review and rewrite your manuscript as it has a similar analysis: Budu, E. Predictors of home births among rural women in Ghana: analysis of data from the 2014 Ghana Demographic and Health Survey. *BMC Pregnancy Childbirth* **20, **523 (2020). https://doi.org/10.1186/s12884-020-03211-4 https://link.springer.com/article/10.1186/s12884-020-03211-4
- The manuscript would benefit from support by a statistical expert to guide on the points mentioned above as well as a review by an editor to ensure the terminology and structure that can facilitate the Reader's understanding. 

==============================

Please submit your revised manuscript by Apr 23 2022 11:59PM. If you will need more time than this to complete your revisions, please reply to this message or contact the journal office at plosone@plos.org. When you're ready to submit your revision, log on to https://www.editorialmanager.com/pone/ and select the 'Submissions Needing Revision' folder to locate your manuscript file.

Please include the following items when submitting your revised manuscript:

- A rebuttal letter that responds to each point raised by the academic editor and reviewer(s). You should upload this letter as a separate file labeled 'Response to Reviewers'.
- A marked-up copy of your manuscript that highlights changes made to the original version. You should upload this as a separate file labeled 'Revised Manuscript with Track Changes'.
- An unmarked version of your revised paper without tracked changes. You should upload this as a separate file labeled 'Manuscript'.

If you would like to make changes to your financial disclosure, please include your updated statement in your cover letter. Guidelines for resubmitting your figure files are available below the reviewer comments at the end of this letter.

Please consider the substantial revisions carefully.  If they can be addressed, we would be pleased to receive your revised manuscript.

Kind regards,

Anayda Portela

Academic Editor

PLOS ONE

Additional Editor Comments (if provided):

see attachment

[Note: HTML markup is below. Please do not edit.]

Reviewers' comments:

Reviewer's Responses to Questions

**Comments to the Author**

1. If the authors have adequately addressed your comments raised in a previous round of review and you feel that this manuscript is now acceptable for publication, you may indicate that here to bypass the “Comments to the Author” section, enter your conflict of interest statement in the “Confidential to Editor” section, and submit your "Accept" recommendation.

Reviewer #1: (No Response)

Reviewer #2: (No Response)

**********

2. Is the manuscript technically sound, and do the data support the conclusions?

The manuscript must describe a technically sound piece of scientific research with data that supports the conclusions. Experiments must have been conducted rigorously, with appropriate controls, replication, and sample sizes. The conclusions must be drawn appropriately based on the data presented. 

Reviewer #1: No

Reviewer #2: No

**********

3. Has the statistical analysis been performed appropriately and rigorously? 

Reviewer #1: Yes

Reviewer #2: Yes

**********

4. Have the authors made all data underlying the findings in their manuscript fully available?

The PLOS Data policy requires authors to make all data underlying the findings described in their manuscript fully available without restriction, with rare exception (please refer to the Data Availability Statement in the manuscript PDF file). The data should be provided as part of the manuscript or its supporting information, or deposited to a public repository. For example, in addition to summary statistics, the data points behind means, medians and variance measures should be available. If there are restrictions on publicly sharing data—e.g. participant privacy or use of data from a third party—those must be specified.

Reviewer #1: Yes

Reviewer #2: No

**********

5. Is the manuscript presented in an intelligible fashion and written in standard English?

PLOS ONE does not copyedit accepted manuscripts, so the language in submitted articles must be clear, correct, and unambiguous. Any typographical or grammatical errors should be corrected at revision, so please note any specific errors here.

Reviewer #1: No

Reviewer #2: Yes

**********

6. Review Comments to the Author

Please use the space provided to explain your answers to the questions above. You may also include additional comments for the author, including concerns about dual publication, research ethics, or publication ethics. (Please upload your review as an attachment if it exceeds 20,000 characters)

Reviewer #1: Thanks to the authors for addressing an important topic and I am glad to review the paper.

The paper is sound interesting and important although lots of work already done on the same topic using secondary DHS dataset. But the novelty of the paper is they have used primary dataset and some important variables have been brought by the authors such transportation facilities, benefits of institutional delivery, fear of child birth at institution.

I am very happy but have some specific thoughts that needs to be addressed for publication and reaching to wider community.

First the methods of the abstract is written generally which is likely very weird for a journal like PLoS one. Please rewrite the methods section.

The ethical approval details with ethical number absent in the paper.

The conclusion of the paper is not specific to the findings.

Why the women don’t have enough information about the importance of institutional delivery, I wonder 83.2% didn’t get the information. Why, the authors are requested to make a recommendations on it.

How good knowledge and poor knowledge and positive and negative attitude measured didn’t clearly discussed , because based on a simple dichotomy question you can not say bad, good bla bla bla.

Rationality of the paper is very week , please focus on the research gaps that is missing in the introduction section.

You have selected some important variables which is impeding women’s to utilise institutional delivery but in title you have used “preferences” it is sound controversial. Because your findings shows women are bound to utilise home delivery then why you have used “Preferences”

I would suggest you to change the title.

Title would be “Why dose the women of Ethiopia bound to utilise home birth?”

Reviewer #2: Comments

1. In the abstract, is missed opportunity to health facility birth synonym to preference to home-birth?

2. In the introduction What is the situation in Ethiopia as far as home birth is concern?

Is all home-birth in Ethiopia assisted by unskilled birth attendant?

Is all health facility birth assisted by skilled attendants?

3. In the method section, have a sub-heading on study population, the inclusion and exclusion criteria

I wonder why did you use pregnant women to answer this research question? Is home-birth recommended in Ethiopia? Does the health system encourage birth preparedness to encourage pregnant women to make plans to allow them to have health facility birth?

Show the calculation on how you arrive to the minimum sample size shown.

It is not clear on how the simple random sampling technique was used as a sampling technique. Did you have the sampling frame, it was a community based study so did you have the address of all women so that if the system picked them you can reach them

The variables and variable measurement section needs clarification, specifically how was the preference to home-birth was measured.

4. In the results, the result on preference among pregnant women is not as well clear, it is indicated in table 2 that only 25.5% of pregnant women were in the position of making decision on their own; Did the study focused in this small proportion of women to answer the research question on preference?

I think this needs to be settled

5. Discussion repeats the results, the explanation to what the key findings mean, the comparison needs explanations as well, similar why, different why. It was difficult to me to follow because of the unanswered question on the outcome variable

6. same to the conclusion

**********

7. PLOS authors have the option to publish the peer review history of their article (what does this mean?). If published, this will include your full peer review and any attached files.

If you choose “no”, your identity will remain anonymous but your review may still be made public.

**Do you want your identity to be public for this peer review?** For information about this choice, including consent withdrawal, please see our Privacy Policy.

Reviewer #1: No

Reviewer #2: **Yes: **Fabiola V. Moshi

[NOTE: If reviewer comments were submitted as an attachment file, they will be attached to this email and accessible via the submission site. Please log into your account, locate the manuscript record, and check for the action link "View Attachments". If this link does not appear, there are no attachment files.]

While revising your submission, please upload your figure files to the Preflight Analysis and Conversion Engine (PACE) digital diagnostic tool, https://pacev2.apexcovantage.com/. PACE helps ensure that figures meet PLOS requirements. To use PACE, you must first register as a user. Registration is free. Then, login and navigate to the UPLOAD tab, where you will find detailed instructions on how to use the tool. If you encounter any issues or have any questions when using PACE, please email PLOS at figures@plos.org. Please note that Supporting Information files do not need this step.

Attachment Submitted filename: Manuscript of preference of Homebirth (4).docx Click here for additional data file.

Attachment Submitted filename: PONE-D-21-39161.pdf Click here for additional data file.

# Author comment: Author response to Decision Letter 1

**DOI:** 10.1371/journal.pone.0276682.r004

25 Mar 2022

RESPONSE TO REVIEWERS # 1

First of all we would like to express our deepest heartfelt thanks to the reviewers for your constructive comments, suggestions and questions to enrich our manuscript. 

� Point raised regarding to the title 

� Responses

� Preference of homebirth is the need of the pregnant mothers to give birth at their home. As they have intention to give birth at their home they may deliver at home which may lead to many life threatening complications. Thus why the title is mandatory to be research question for the identification of its prevalence and associated factors.

� In Ethiopian context, homebirth is not allowed for women instead going health facility for giving birth is the first priority. The reason is that home delivery is leading to many complications since it is not being conducted by skilled birth attendants but still now many pregnant mothers prefer to give birth at their home ignoring all life threatening complications. Thus, this research was aimed to identify prevalence and factors associated with this preference of homebirth.

� Points raised in Abstract

� Responses

� Missed opportunities changed to preferring homebirth because of the intention was talking about losing the care being given in health institutions since they preferred giving birth at home rather than health institutions.

� Study area Arba Minch zuria woreda surveillance site.

� The data were primary for this research and directly collected from the study participants.

� Negative attitude towards services and high fear to give birth at institution are two different independent variables which were independently assessed in multivariable binary logistic regression to identify association with preference of homebirth.

� Candidate variables in bi-variable binary logistic regression at p-value < 0.25 were transferred to multivariable binary logistic regression according to the rule of hosmer and lemishew as the model was fitted with hosmer and lemishew goodness of fit to identify the variables significantly associated with preference of homebirth.

� Measurements of preference of homebirth was obtained from the question asked to pregnant women; “where did you prefer to give birth?” Response to this question was either of home birth or health facility [hospital, health Centre/clinic, health post, and private hospital/clinic.

� The reason why such question is used for measurement of preference of place of birth preference in rural part of the Ethiopia starting from the traditional view to a now days left with the pregnant mother that they give birth at their preference area due to they are not flexible to the service being given at health institution thinking that traditional birth attendants give better services than health professionals. Additionally, this study was about the place preferred by pregnant mother for giving birth not about delivered birth place. 

� Study participants were 416 pregnant mothers

� Points raised about measurement of outcome variable (preference of home birth) 

� Responses

� The outcome variable “preference of homebirth” was obtained from the question asked to pregnant women; “where do you prefer to give birth?” Response to this question was either of home birth or health facility (hospital, health Centre/clinic, health post, and private hospital/clinic). Preference of homebirth is not equivalent with home delivery because it is only about the intention (preference) of the pregnant mothers want to give birth at their own home rather than preferring to deliver at health institutions. Therefore this outcome variable was only about the need of the pregnant mothers to give birth at their home.

� Points raised regarding to justification of the study 

� Responses

� No study done on the preference of home birth among pregnant women in rural areas. Additionally, this study addressed different variables (transportation facilities, benefits of institutional delivery, fear of child birth at institution) those were not addressed by previous studies.

� Points raised regarding to assessment of knowledge and attitude 

� Responses

� Knowledge about danger signs of pregnancy, labor, and following childbirth: Knowledge about danger sign was assessed based on the women’s response to eight knowledge questions. Thus, women’s were considered as they have good knowledge if they answered correctly to four or more knowledge question.

� Women’s Attitude about skilled birth services: A total of 7 questions were used to assess attitude. Women responded to each question in the form of very agree, agree, disagree, and very disagree. Very agree and Agree was labeled as value "1", and disagree and very disagree was as assigned value "0". Women were considered as they have positive attitudes if all questions were labeled a value "1", and negative attitudes if any of the questions are labeled "0"

� Points raised regarding to reference number of Ethical clearance 

� Responses 

� An ethical clearance letter was obtained from Arba Minch University, college of medicine, and health sciences research ethics review board in 25/03/2021 with reference number IRB/1071/21. 

� Points raised regarding to conclusions: 

� The preference of home birth is 24% among pregnant women in the study area. Husband involvement in decision making, no access of road for transportation, not heard about the benefit of institutional birth, poor knowledge about danger signs, negative attitude toward services, and high fear to give birth at health institutions were factors significantly associated with the preference of home birth. 

RESPONSE TO REVIEWERS # 2

First of all we would like to express our deepest heartfelt thanks to reviewer # 2 for your constructive comments, suggestions and questions to enrich our manuscript. 

� Points raised in Abstract

� Responses

� Missed opportunities changed to preferring homebirth because of the intention was talking about losing the care being given in health institutions since they preferred giving birth at home rather than health institutions.

� Study area Arba Minch zuria woreda surveillance site.

� The data were primary for this research and directly collected from the study participants.

� Negative attitude towards services and high fear to give birth at institution are two different independent variables which were independently assessed in multivariable binary logistic regression to identify association with preference of homebirth.

� Candidate variables in bi-variable binary logistic regression at p-value < 0.25 were transferred to multivariable binary logistic regression according to the rule of hosmer and lemishew as the model was fitted with hosmer and lemishew goodness of fit to identify the variables significantly associated with preference of homebirth.

� Measurements of preference of homebirth was obtained from the question asked to pregnant women; “where did you prefer to give birth?” Response to this question was either of home birth or health facility [hospital, health Centre/clinic, health post, and private hospital/clinic.

� The reason why such question is used for measurement of preference of place of birth preference in rural part of the Ethiopia starting from the traditional view to a now days left with the pregnant mother that they give birth at their preference area due to they are not flexible to the service being given at health institution thinking that traditional birth attendants give better services than health professionals. Additionally, this study was about the place preferred by pregnant mother for giving birth not about delivered birth place. 

� Study participants were 416 pregnant mothers

� Points raised about measurement of outcome variable (preference of home birth) 

� Responses

� The outcome variable “preference of homebirth” was obtained from the question asked to pregnant women; “where do you prefer to give birth?” Response to this question was either of home birth or health facility (hospital, health Centre/clinic, health post, and private hospital/clinic). Preference of homebirth is not equivalent with home delivery because it is only about the intention (preference) of the pregnant mothers want to give birth at their own home rather than preferring to deliver at health institutions. Therefore this outcome variable was only about the need of the pregnant mothers to give birth at their home.

� Measurements of variables

� Responses

� Knowledge about danger signs of pregnancy, labor, and following childbirth: Knowledge about danger sign was assessed based on the women’s response to eight knowledge questions. Thus, women’s were considered as they have good knowledge if they answered correctly to four or more knowledge question.

� Women’s Attitude about skilled birth services: A total of 7 questions were used to assess attitude. Women responded to each question in the form of very agree, agree, disagree, and very disagree. Very agree and Agree was labeled as value "1", and disagree and very disagree was as assigned value "0". Women were considered as they have positive attitudes if all questions were labeled a value "1", and negative attitudes if any of the questions are labeled "0"

� Points raised regarding to situation of Home birth Ethiopia 

� Responses

� According to mini Ethiopia public health 2019 report half of pregnant women in Ethiopia gave birth at home.

� All most all home delivery In Ethiopia attended without skilled birth attendants. In addition to this majority of Ethiopian population live in rural areas with lack of infrastructure including health sectors.

� Now a day facility delivery service provided by skilled birth attendants accredited from known university or college.

� Points raised regarding to why study participant pregnant women is

� Responses

� It is necessary to find out their needs and factors that affect care-seeking behavior in the given context. Because when they prefer to give birth at their home the probability of giving birth at institutions is very low since their internal need is not to delivery at health facilities, maternal mortality may follow the consequence. 

� Women may give birth at home due to this; complications can occur during labor and delivery that is why we select the pregnant women as study participant. Then identifying the problem helps to intervene and create awareness that may come to their life due preference of homebirth. 

� Points raised regarding to techniques of sampling for this study 

� Responses

� Hence study was conducted in Arba Minch health and demographic surveillance site the list of pregnant women was obtained from health extension workers working in Arba Minch health and demographic surveillance. So the sample frame was obtained from the health extension workers in the surveillance site.

Attachment Submitted filename: RESPONSE TO REVIEWERS.docx Click here for additional data file.

# Peer review: Decision Letter 2

**DOI:** 10.1371/journal.pone.0276682.r005

**Authors:** Steve Zimmerman

23 Jun 2022

PONE-D-21-39161R2

PREFERENCE OF HOMEBIRTH AND ASSOCIATED FACTORS AMONG PREGNANT WOMEN IN ARBA MINCH HEALTH AND DEMOGRAPHIC SURVEILLANCE SITE, SOUTHERN ETHIOPIA

PLOS ONE

Dear Dr. Feyisa,

Thank you for submitting your manuscript to PLOS ONE. After careful consideration, we feel that it has merit but does not fully meet PLOS ONE’s publication criteria as it currently stands. Therefore, we invite you to submit a revised version of the manuscript that addresses the points raised during the review process.

Please see the comments made by reviewer #2 below, and look at the comments and suggestions in the attached documents. In addition, there were requests made by the Academic Editor that I do not think you addressed in this last revision.

Specifically:

"(1) There are several concerns regarding the methods and a description of the methods.  The article appears to be a secondary data analysis but this is not stated.  The study variables, particularly the outcome variable, are not clearly explained; the reviewers were confused as to the outcome used in your model. Only the variables that showed statistical significance in the bivariate analysis should be used for the regression analysis.  

(2) I refer you to an article that may be useful to you as you review and rewrite your manuscript as it has a similar analysis: Budu, E. Predictors of home births among rural women in Ghana: analysis of data from the 2014 Ghana Demographic and Health Survey. BMC Pregnancy Childbirth 20, 523 (2020). https://doi.org/10.1186/s12884-020-03211-4 https://link.springer.com/article/10.1186/s12884-020-03211-4  

(3) The manuscript would benefit from support by a statistical expert to guide on the points mentioned above as well as a review by an editor to ensure the terminology and structure that can facilitate the Reader's understanding."

Please address these requests in your revised manuscript.

Please submit your revised manuscript by Aug 06 2022 11:59PM. If you will need more time than this to complete your revisions, please reply to this message or contact the journal office at plosone@plos.org. When you're ready to submit your revision, log on to https://www.editorialmanager.com/pone/ and select the 'Submissions Needing Revision' folder to locate your manuscript file.

Please include the following items when submitting your revised manuscript:

- A rebuttal letter that responds to each point raised by the academic editor and reviewer(s). You should upload this letter as a separate file labeled 'Response to Reviewers'.
- A marked-up copy of your manuscript that highlights changes made to the original version. You should upload this as a separate file labeled 'Revised Manuscript with Track Changes'.
- An unmarked version of your revised paper without tracked changes. You should upload this as a separate file labeled 'Manuscript'.

If you would like to make changes to your financial disclosure, please include your updated statement in your cover letter. Guidelines for resubmitting your figure files are available below the reviewer comments at the end of this letter.

If applicable, we recommend that you deposit your laboratory protocols in protocols.io to enhance the reproducibility of your results. Protocols.io assigns your protocol its own identifier (DOI) so that it can be cited independently in the future. For instructions see: https://journals.plos.org/plosone/s/submission-guidelines#loc-laboratory-protocols. Additionally, PLOS ONE offers an option for publishing peer-reviewed Lab Protocol articles, which describe protocols hosted on protocols.io. Read more information on sharing protocols at https://plos.org/protocols?utm_medium=editorial-email&utm_source=authorletters&utm_campaign=protocols.

We look forward to receiving your revised manuscript.

Kind regards,

Steve Zimmerman, PhD

Associate Editor, PLOS ONE

[Note: HTML markup is below. Please do not edit.]

Reviewers' comments:

Reviewer's Responses to Questions

**Comments to the Author**

1. If the authors have adequately addressed your comments raised in a previous round of review and you feel that this manuscript is now acceptable for publication, you may indicate that here to bypass the “Comments to the Author” section, enter your conflict of interest statement in the “Confidential to Editor” section, and submit your "Accept" recommendation.

Reviewer #1: (No Response)

Reviewer #2: All comments have been addressed

**********

2. Is the manuscript technically sound, and do the data support the conclusions?

The manuscript must describe a technically sound piece of scientific research with data that supports the conclusions. Experiments must have been conducted rigorously, with appropriate controls, replication, and sample sizes. The conclusions must be drawn appropriately based on the data presented. 

Reviewer #1: Partly

Reviewer #2: Yes

**********

3. Has the statistical analysis been performed appropriately and rigorously? 

Reviewer #1: Yes

Reviewer #2: Yes

**********

4. Have the authors made all data underlying the findings in their manuscript fully available?

The PLOS Data policy requires authors to make all data underlying the findings described in their manuscript fully available without restriction, with rare exception (please refer to the Data Availability Statement in the manuscript PDF file). The data should be provided as part of the manuscript or its supporting information, or deposited to a public repository. For example, in addition to summary statistics, the data points behind means, medians and variance measures should be available. If there are restrictions on publicly sharing data—e.g. participant privacy or use of data from a third party—those must be specified.

Reviewer #1: No

Reviewer #2: No

**********

5. Is the manuscript presented in an intelligible fashion and written in standard English?

PLOS ONE does not copyedit accepted manuscripts, so the language in submitted articles must be clear, correct, and unambiguous. Any typographical or grammatical errors should be corrected at revision, so please note any specific errors here.

Reviewer #1: Yes

Reviewer #2: Yes

**********

6. Review Comments to the Author

Please use the space provided to explain your answers to the questions above. You may also include additional comments for the author, including concerns about dual publication, research ethics, or publication ethics. (Please upload your review as an attachment if it exceeds 20,000 characters)

Reviewer #1: Thanks for the invitation to review the paper.

Already similar studies are available this study is not caring any important findings for public health literature.

Even the analysis is not robust and not standard for high quality journal like PLoS one. I am recommending submission to elsewhere similar journals.

Reviewer #2: Authors have addressed the raised comments satisfactorily but some areas need revisions

1. the first sentence of conclusion of abstract stand like it is reported in the results part. I suggest in the conclusion to state what does the found 24% mean, which message does it carry.

2. the method section still lacks clarity, in the first review i suggested to separate the subheadings so that a reader can follow, study design and setting stand together but other sub-headings such as sampling technique and others need their own sub-heading, so I suggest authors to rework on the section

3. the sampling techniques stated was used in the study still not well stated, authors have to explain clearly how was it possible for them to do random sampling in a community based study

4. Variable measurement section is needed in the method, mention the variables which were used and how were they measured

5. The factors associated with preference is not adequately discusses, the big share of the discussion is carried with the descriptive findings and the factors are not adequately discussed

4. Assessment of language is needed, data always go with were but some areas it is mentioned data was.

**********

7. PLOS authors have the option to publish the peer review history of their article (what does this mean?). If published, this will include your full peer review and any attached files.

If you choose “no”, your identity will remain anonymous but your review may still be made public.

**Do you want your identity to be public for this peer review?** For information about this choice, including consent withdrawal, please see our Privacy Policy.

Reviewer #1: No

Reviewer #2: **Yes: **Fabiola Moshi

**********

[NOTE: If reviewer comments were submitted as an attachment file, they will be attached to this email and accessible via the submission site. Please log into your account, locate the manuscript record, and check for the action link "View Attachments". If this link does not appear, there are no attachment files.]

While revising your submission, please upload your figure files to the Preflight Analysis and Conversion Engine (PACE) digital diagnostic tool, https://pacev2.apexcovantage.com/. PACE helps ensure that figures meet PLOS requirements. To use PACE, you must first register as a user. Registration is free. Then, login and navigate to the UPLOAD tab, where you will find detailed instructions on how to use the tool. If you encounter any issues or have any questions when using PACE, please email PLOS at figures@plos.org. Please note that Supporting Information files do not need this step.

Attachment Submitted filename: MANUSCRIPT OF HOME BIRTH PREFERENCE (1).docx Click here for additional data file.

# Author comment: Author response to Decision Letter 2

**DOI:** 10.1371/journal.pone.0276682.r006

30 Jun 2022

RESPONSE TO EDITOR 

First of all we would like to express our deepest heartfelt thanks to the editor for your constructive comments, suggestions and questions to enrich our manuscript. 

� Point raised regarding to the article appears to be a secondary data analysis

� Responses

� The study entitled with “Preference of homebirth and associated factors among pregnant women in Arba Minch health and demographic surveillance site, southern Ethiopia” was conducted by collecting primary data from selected pregnant women of Arba Minch health and demographic surveillance site. Therefore the title was the research of primary data

� The data were primary for this research and directly collected from the study participants.

� Preference of homebirth is the need of the pregnant mothers to give birth at their home. As they have intention to give birth at their home they may deliver at home which may lead to many life threatening complications. Thus why the title is mandatory to be research question for the identification of its prevalence and associated factors.

� In Ethiopian context, homebirth is not allowed for women instead going health facility for giving birth is the first priority. The reason is that home delivery is leading to many complications since it is not being conducted by skilled birth attendants but still now many pregnant mothers prefer to give birth at their home ignoring all life threatening complications. Thus, this research was aimed to identify prevalence and factors associated of preference of homebirth using primary data collected.

� Points raised regarding to variables in regression

� Responses

 Study variables 

� Dependent variable 

� Preference of home birth 

� “Preference of home birth” was the dependent variable and was obtained from the question, “Where do you prefer/need to give birth [choices]?” Response to this question was prefer/need to give birth at home or at government hospital/health center or private hospital/clinic. It was then dichotomized to into prefer facility birth = 0 and prefer home birth = 1 where respondent’s preference/need to give birth at home “prefer home birth” and all the other categories were grouped as “prefer facility birth”. 

� Independent variables 

� The independent variables considered in this study were age of the women, marital status, ethnicity, religion, women educational status, women occupation, husband educational status, husband occupation, household income, residence, family size, Gravid, pregnancy desire, last place of delivery, last mode of delivery, last birth complication, current ANC status, number of ANC follow up, birth interval, distance from health services/facility, road access for transportation to health institutions, information on the benefit of institutional birth, Knowledge of danger signs, attitude toward skilled birth services, decision-making, and fear of childbirth at the institution

� Ten (10) candidate variables in bi-variable binary logistic regression at p-value < 0.25 were transferred to multivariable binary logistic regression according to the rule of hosmer and lemishew as the model was fitted with hosmer and lemishew goodness of fit to identify the variables significantly associated with preference of homebirth.

� Points raised regarding to description of the methods specifically Study area, study design, and study population, data collection period and study variables to be described using separate sub-headings

� Response

� Separately described in manuscript according to reviewer enquire of sub-headings separately.

RESPONSE TO REVIEWER # 2

First of all we would like to express our deepest heartfelt thanks to reviewer # 2 for your constructive comments, suggestions and questions to enrich our manuscript. 

� Point raised regarding to the conclusion to state what does the found 24% mean? 

� Response

� This indicates that in this study, in Arba Minch demographic health surveillance site, the prevalence of the need (preference) of pregnant women to give birth at their home was 24% [95%CI: (19.9%-28.2%)].

� Point raised regarding to separate the subheadings so that a reader can follow, study design and setting stand together but other sub-headings such as sampling technique and others need their own sub-heading

� Responses

� Separately done as per of the enquiry 

� Study design and study area

A community-based cross-sectional study was conducted in Arba Minch Health and Demographic Surveillance Site. Arba Minch Health and Demographic Surveillance Site are located in Arba Minch Zuria and Gacho Baba districts, Gamo Zone, Southern Ethiopia, 500 km to the South of Addis Ababa, the capital city of Ethiopia. Arba Minch Zuria district and Gacho Baba district had a total of 31 kebeles [smallest administrative units] and it is included under Arba Minch Zuria Demographic and Health Development Program (AM-DHDP). AM-DHDP is owned by Arba Minch University and it is one of the six public universities Health and Demographic Surveillance System (HDSS) in Ethiopia. The surveillance site consists of nine kebeles which were selected in the representation of 31 kebeles in the district. From them, 6 kebeles were found in Arba Minch zuria district, and the rest three were found in Gacho baba districts. Farming is the predominant occupation of residents in the districts. Based on the 2007 census projection, the districts had a total population of 164,529. The district has 7 health centers and 37 health posts. Around 81.8% of women gave birth at home in Arba Minch Zuria district.

� Data collection period

Data were collected from May 1- June 1, 2021 from randomly selected pregnant women of Arba Minch zuria woreda.

� Study population

Pregnant women living in selected nine Kebeles of Arba Minch health and demographic surveillance site were study population for this study.

� Points raised regarding to techniques of how was it possible for them to do random sampling in a community based study

� Responses

� Hence study was conducted in Arba Minch health and demographic surveillance site registration and identification of the women becoming pregnant with their address is one of the core and continuum activities of the health extension workers assigned to the woreda. As well registration and identification of the women becoming pregnant with their address are common activity in all parts of Ethiopia. Thus lists of pregnant women were obtained from health extension workers working in Arba Minch health and demographic surveillance. So the sampling frames were obtained from the health extension workers in the surveillance site then simple random sampling technique was applied using computer random generated numbers.

� Points raised regarding to Variable measurement section is needed in the method

� Response

� Modification was done as follows in the manuscript

� Study variables 

� Dependent variable 

� Preference of home birth 

� “Preference of home birth” was the dependent variable and was obtained from the question, “Where do you prefer/need to give birth [choices]?” Response to this question was prefer/need to give birth at home or at government hospital/health center or private hospital/clinic. It was then dichotomized to into prefer facility birth = 0 and prefer home birth = 1 where respondent’s preference/need to give birth at home “prefer home birth” and all the other categories were grouped as “prefer facility birth” [11].

� Independent variables 

� The independent variables considered in this study were age of the women, marital status, ethnicity, religion, women educational status, women occupation, husband educational status, husband occupation, household income, residence, family size, Gravid, pregnancy desire, last place of delivery, last mode of delivery, last birth complication, current ANC status, number of ANC follow up, birth interval, distance from health services/facility, road access for transportation to health institutions, information on the benefit of institutional birth, Knowledge of danger signs, attitude toward skilled birth services, decision-making, and fear of childbirth at the institution 

� Operational definitions

� Women’s fear of childbirth at health institution: A total of 13 items were presented to assess fear of childbirth at the health institution. Women responded to their level of fear for each item by a 4-point Likert scale. The women were classified as high fear if they scored mean value and above, and low fear if they scored less than mean value to question assessing fear of childbirth at institutions [19].

� Knowledge about danger signs of pregnancy, labor, and following childbirth: Knowledge about danger sign was assessed based on the women’s response to eight knowledge questions. Thus, women’s were considered as they have good knowledge if they answered correctly to four or more knowledge question [20].

� Women’s Attitude about skilled birth services: A total of 7 questions were used to assess attitude. Women responded to each question in the form of very agree, agree, disagree, and very disagree. Very agree and Agree was labeled as value "1", and disagree and very disagree was as assigned value "0". Women were considered as they have positive attitudes if all questions were labeled a value "1", and negative attitudes if any of the questions are labeled "0" [17] 

� Point raised regarding to discussion and language edition

� Response 

� Modification were made to enrich discussion part especially factor related part.

� “Was” changed to “were” following the word data.

Attachment Submitted filename: RESPONSE TO REVIEWERS.docx Click here for additional data file.

# Peer review: Decision Letter 3

**DOI:** 10.1371/journal.pone.0276682.r007

**Authors:** Frank T. Spradley

19 Sep 2022

PONE-D-21-39161R3PREFERENCE OF HOMEBIRTH AND ASSOCIATED FACTORS AMONG PREGNANT WOMEN IN ARBA MINCH HEALTH AND DEMOGRAPHIC SURVEILLANCE SITE, SOUTHERN ETHIOPIAPLOS ONE

Dear Dr. Feyisa,

Thank you for submitting your manuscript to PLOS ONE. After careful consideration, we feel that it has merit but does not fully meet PLOS ONE’s publication criteria as it currently stands. Therefore, we invite you to submit a revised version of the manuscript that addresses the points raised during the review process.

Please submit your revised manuscript by Nov 03 2022 11:59PM. If you will need more time than this to complete your revisions, please reply to this message or contact the journal office at plosone@plos.org. When you're ready to submit your revision, log on to https://www.editorialmanager.com/pone/ and select the 'Submissions Needing Revision' folder to locate your manuscript file.

Please include the following items when submitting your revised manuscript:

- A rebuttal letter that responds to each point raised by the academic editor and reviewer(s). You should upload this letter as a separate file labeled 'Response to Reviewers'.
- A marked-up copy of your manuscript that highlights changes made to the original version. You should upload this as a separate file labeled 'Revised Manuscript with Track Changes'.
- An unmarked version of your revised paper without tracked changes. You should upload this as a separate file labeled 'Manuscript'.

If you would like to make changes to your financial disclosure, please include your updated statement in your cover letter. Guidelines for resubmitting your figure files are available below the reviewer comments at the end of this letter.

If applicable, we recommend that you deposit your laboratory protocols in protocols.io to enhance the reproducibility of your results. Protocols.io assigns your protocol its own identifier (DOI) so that it can be cited independently in the future. For instructions see: https://journals.plos.org/plosone/s/submission-guidelines#loc-laboratory-protocols. Additionally, PLOS ONE offers an option for publishing peer-reviewed Lab Protocol articles, which describe protocols hosted on protocols.io. Read more information on sharing protocols at https://plos.org/protocols?utm_medium=editorial-email&utm_source=authorletters&utm_campaign=protocols.

We look forward to receiving your revised manuscript.

Kind regards,

Frank T. Spradley

Academic Editor

PLOS ONE

Journal Requirements:

Please review your reference list to ensure that it is complete and correct. If you have cited papers that have been retracted, please include the rationale for doing so in the manuscript text, or remove these references and replace them with relevant current references. Any changes to the reference list should be mentioned in the rebuttal letter that accompanies your revised manuscript. If you need to cite a retracted article, indicate the article’s retracted status in the References list and also include a citation and full reference for the retraction notice.

[Note: HTML markup is below. Please do not edit.]

Reviewers' comments:

Reviewer's Responses to Questions

**Comments to the Author**

1. If the authors have adequately addressed your comments raised in a previous round of review and you feel that this manuscript is now acceptable for publication, you may indicate that here to bypass the “Comments to the Author” section, enter your conflict of interest statement in the “Confidential to Editor” section, and submit your "Accept" recommendation.

Reviewer #2: All comments have been addressed

**********

2. Is the manuscript technically sound, and do the data support the conclusions?

The manuscript must describe a technically sound piece of scientific research with data that supports the conclusions. Experiments must have been conducted rigorously, with appropriate controls, replication, and sample sizes. The conclusions must be drawn appropriately based on the data presented. 

Reviewer #2: Yes

**********

3. Has the statistical analysis been performed appropriately and rigorously? 

Reviewer #2: Yes

**********

4. Have the authors made all data underlying the findings in their manuscript fully available?

The PLOS Data policy requires authors to make all data underlying the findings described in their manuscript fully available without restriction, with rare exception (please refer to the Data Availability Statement in the manuscript PDF file). The data should be provided as part of the manuscript or its supporting information, or deposited to a public repository. For example, in addition to summary statistics, the data points behind means, medians and variance measures should be available. If there are restrictions on publicly sharing data—e.g. participant privacy or use of data from a third party—those must be specified.

Reviewer #2: No

**********

5. Is the manuscript presented in an intelligible fashion and written in standard English?

PLOS ONE does not copyedit accepted manuscripts, so the language in submitted articles must be clear, correct, and unambiguous. Any typographical or grammatical errors should be corrected at revision, so please note any specific errors here.

Reviewer #2: Yes

**********

6. Review Comments to the Author

Please use the space provided to explain your answers to the questions above. You may also include additional comments for the author, including concerns about dual publication, research ethics, or publication ethics. (Please upload your review as an attachment if it exceeds 20,000 characters)

Reviewer #2: The manuscript is improved with minor corrections

1. In the background information, include other factors associated with choices of place for childbirth as reported in the literature

2. Sampling technique is not well elaborated, how did you ensure probability sampling

3. In the data collection I suggest you change the use of face to face interview to interviewer administered questionnaire because mostly face to face interview is used in qualitative studies

**********

7. PLOS authors have the option to publish the peer review history of their article (what does this mean?). If published, this will include your full peer review and any attached files.

If you choose “no”, your identity will remain anonymous but your review may still be made public.

**Do you want your identity to be public for this peer review?** For information about this choice, including consent withdrawal, please see our Privacy Policy.

Reviewer #2: **Yes: **Fabiola Moshi

**********

[NOTE: If reviewer comments were submitted as an attachment file, they will be attached to this email and accessible via the submission site. Please log into your account, locate the manuscript record, and check for the action link "View Attachments". If this link does not appear, there are no attachment files.]

While revising your submission, please upload your figure files to the Preflight Analysis and Conversion Engine (PACE) digital diagnostic tool, https://pacev2.apexcovantage.com/. PACE helps ensure that figures meet PLOS requirements. To use PACE, you must first register as a user. Registration is free. Then, login and navigate to the UPLOAD tab, where you will find detailed instructions on how to use the tool. If you encounter any issues or have any questions when using PACE, please email PLOS at figures@plos.org. Please note that Supporting Information files do not need this step.

# Author comment: Author response to Decision Letter 3

**DOI:** 10.1371/journal.pone.0276682.r008

4 Oct 2022

RESPONSE TO REVIEWER # 2

First of all we would like to express our deepest heartfelt thanks to reviewer # 2 for your constructive comments, suggestions and questions to enrich our manuscript. 

� In the background information, include other factors associated with choices of place for childbirth as reported in the literature

� Response

� As one of the components should be explained in the introduction part the common factors contributed to the preference of home birth from the literatures were age of the pregnant mothers, Lack of knowledge on danger sign, poor road access, lack of ANC follow-up, low household income, place of last delivery, parity, and low educational status (11-13).

� Sampling technique is not well elaborated, how did you ensure probability sampling

� Responses

� Simple random probability sampling technique was used in this study to ensure the representativeness of the information because lists of pregnant women is available as every pregnant mother in every district and Kebeles should be registered by health extension workers of Ethiopia. Thus why their data which contains these pregnant mothers is always available and researcher can get the lists and use for scientific purposes. Therefore, simple random sampling technique was ensured due to the lists/sampling frame of the pregnant mother of the study area were available.

� Hence study was conducted in Arba Minch health and demographic surveillance site registration and identification of the women becoming pregnant with their address is one of the core and continuum activities of the health extension workers assigned to the woreda. As well registration and identification of the women becoming pregnant with their address are common activity in all parts of Ethiopia. Thus lists of pregnant women were obtained from health extension workers working in Arba Minch health and demographic surveillance. So the sampling frames were obtained from the health extension workers in the surveillance site then simple random sampling technique was applied using computer random generated numbers.

� In the data collection I suggest you change the use of face to face interview to interviewer administered questionnaire because mostly face to face interview is used in qualitative studies

� Responses

� As per the recommendation face to face interview is changed to “interviewer-administered questionnaire” in the manuscript.

Attachment Submitted filename: RESPONSE TO REVIEWERS.docx Click here for additional data file.

# Editor report: Decision Letter 4

**DOI:** 10.1371/journal.pone.0276682.r009

**Authors:** Frank T. Spradley

12 Oct 2022

PREFERENCE OF HOMEBIRTH AND ASSOCIATED FACTORS AMONG PREGNANT WOMEN IN ARBA MINCH HEALTH AND DEMOGRAPHIC SURVEILLANCE SITE, SOUTHERN ETHIOPIA

PONE-D-21-39161R4

Dear Dr. Feyisa,

We’re pleased to inform you that your manuscript has been judged scientifically suitable for publication and will be formally accepted for publication once it meets all outstanding technical requirements.

Within one week, you’ll receive an e-mail detailing the required amendments. When these have been addressed, you’ll receive a formal acceptance letter and your manuscript will be scheduled for publication.

An invoice for payment will follow shortly after the formal acceptance. To ensure an efficient process, please log into Editorial Manager at http://www.editorialmanager.com/pone/, click the 'Update My Information' link at the top of the page, and double check that your user information is up-to-date. If you have any billing related questions, please contact our Author Billing department directly at authorbilling@plos.org.

If your institution or institutions have a press office, please notify them about your upcoming paper to help maximize its impact. If they’ll be preparing press materials, please inform our press team as soon as possible -- no later than 48 hours after receiving the formal acceptance. Your manuscript will remain under strict press embargo until 2 pm Eastern Time on the date of publication. For more information, please contact onepress@plos.org.

Kind regards,

Frank T. Spradley

Academic Editor

PLOS ONE

# Editor report: Acceptance letter

**DOI:** 10.1371/journal.pone.0276682.r010

**Authors:** Frank T. Spradley

20 Oct 2022

PONE-D-21-39161R4 

Preference of homebirth and associated factors among pregnant women in Arba Minch health and demographic surveillance site, Southern Ethiopia 

Dear Dr. Feyisa:

I'm pleased to inform you that your manuscript has been deemed suitable for publication in PLOS ONE. Congratulations! Your manuscript is now with our production department. 

If your institution or institutions have a press office, please let them know about your upcoming paper now to help maximize its impact. If they'll be preparing press materials, please inform our press team within the next 48 hours. Your manuscript will remain under strict press embargo until 2 pm Eastern Time on the date of publication. For more information please contact onepress@plos.org.

If we can help with anything else, please email us at plosone@plos.org. 

Thank you for submitting your work to PLOS ONE and supporting open access. 

Kind regards, 

PLOS ONE Editorial Office Staff

on behalf of

Dr. Frank T. Spradley 

Academic Editor

PLOS ONE
